# Cell-Cell Transmission Enables HIV-1 to Evade Inhibition by Potent CD4bs Directed Antibodies

**DOI:** 10.1371/journal.ppat.1002634

**Published:** 2012-04-05

**Authors:** Irene A. Abela, Livia Berlinger, Merle Schanz, Lucy Reynell, Huldrych F. Günthard, Peter Rusert, Alexandra Trkola

**Affiliations:** 1 Institute of Medical Virology, University of Zurich, Zurich, Switzerland; 2 PhD Program in Microbiology and Immunology, University of Zurich, Zurich, Switzerland; 3 MD-PhD Program, University of Zurich, Zurich, Switzerland; 4 Division of Infectious Diseases, University Hospital Zurich, University of Zurich, Zurich, Switzerland; Harvard University, United States of America

## Abstract

HIV is known to spread efficiently both in a cell-free state and from cell to cell, however the relative importance of the cell-cell transmission mode in natural infection has not yet been resolved. Likewise to what extent cell-cell transmission is vulnerable to inhibition by neutralizing antibodies and entry inhibitors remains to be determined. Here we report on neutralizing antibody activity during cell-cell transmission using specifically tailored experimental strategies which enable unambiguous discrimination between the two transmission routes. We demonstrate that the activity of neutralizing monoclonal antibodies (mAbs) and entry inhibitors during cell-cell transmission varies depending on their mode of action. While gp41 directed agents remain active, CD4 binding site (CD4bs) directed inhibitors, including the potent neutralizing mAb VRC01, dramatically lose potency during cell-cell transmission. This implies that CD4bs mAbs act preferentially through blocking free virus transmission, while still allowing HIV to spread through cell-cell contacts. Thus providing a plausible explanation for how HIV maintains infectivity and rapidly escapes potent and broadly active CD4bs directed antibody responses *in vivo*.

## Author Summary

HIV is known to spread both in a cell-free state and from cell to cell, however the relative importance of the cell-cell transmission mode in natural infection has not yet been resolved. Design of vaccines attempt to inhibit HIV entry into target cells as do engineered entry inhibitors used as therapeutics. While these agents are known to block the entry of cell-free HIV particles into cells, to what extent cell-cell transmission is vulnerable to such inhibition is unclear. Here we report that the activity of neutralizing antibodies and inhibitors during cell-cell transmission varies depending on their mode of action. A prominent class of neutralizing antibodies directed to the CD4 binding site on the virus envelope very efficiently blocks binding of the virus to its primary receptor on target cells, the CD4 molecule. These types of antibodies are elicited in natural infection and once isolated from infected individuals have shown to be highly potent. Why HIV still replicates in the presence of such potent antibodies remains unclear. Here we show that these CD4 binding site antibodies are dramatically less potent inhibitors of cell-cell transmission, and therefore act preferentially by blocking free virus transmission while allowing HIV to spread through cell-cell contact.

## Introduction

The Human Immunodeficiency Virus (HIV) spreads *in vitro* very efficiently, if not preferentially, by cell-cell contacts. Viral transmission from infected to non-infected cells occurs via formation of virological synapses – organized contact areas which concentrate cellular entry receptors and virions [1], [2], [3], [4], [5] - and via transient cell-cell contacts and longer-range intercellular interactions including nanotubes and filopodia [6], [7], [8]. Virus transmission through these points of contiguity has been proven *in vitro* to be more efficient and rapid than infection by cell-free viruses [9], [10], [11], [12], [13], [14], supporting the notion that cell-cell transmission may be a relevant if not dominant mode of virus dissemination in infected individuals. The highly efficient transmission of HIV between cells may also foster infection of target cells with multiple virions and so facilitate recombination and escape adaptations to occur more frequently [15], [16], [17], [18]. So far the relative contribution of cell-cell and cell-free virus transmission in acquisition of HIV infection and viral dissemination during human infection remain however undefined. This gap in knowledge poses a conceptual problem for neutralizing antibody based HIV vaccine and entry inhibitor design, as it remains uncertain whether both cell-free and cell-cell spread of HIV must be blocked with equal efficacy, or whether only the dominant transmission mode needs to be targeted and if so which.

Neutralizing antibodies recognize epitopes on the envelope glycoproteins gp120 and gp41 that are accessible in the oligomeric form of the HIV envelope protein [19], [20]. Neutralization occurs by blocking virion attachment to host cell receptors or by inhibiting membrane fusion [19]. To date it remains unclear to what extent the relatively enclosed environment of the viral synapse is able to protect the virus from humoral immunity [21], [22]. Previous attempts to determine the capacity of individual neutralizing antibodies to inhibit cell-cell transmission came to varying and conflicting conclusions, suggesting it was entirely inefficient, less efficient than inhibition of cell-free infection, or indeed equally efficient than inhibition of cell-free infection [13], [22], [23], [24], [25], [26]. These discrepancies in reported neutralizing antibody efficacy in blocking HIV cell-cell transmission underline the complexity of studying HIV transmission modes and were suggested to likely reflect incongruities amongst cell types studied as well as differences in experimental procedures [21]. A number of studies have shed light on the complexity of HIV transmission modes and revealed substantial differences amongst experimental set ups used to study cell-cell transmission [5], [13], [21], [22], [27]. Cell-associated HIV can be transmitted to uninfected target cells by a variety of modes and may involve both, cells that are productively infected (cis-infection) and cells that trapped virus but remained uninfected (trans-infection [28], [29], [30]. Depending on the cell type of the counter partners, their relative frequencies and rate of infection, transmission events can differ on a molecular level and were described to depend on a range of extracellular interaction structures (T-T cell viral synapse [4], DC-T-cell viral synapse [3], Macrophage-T-cell [31], polysynapses [7], nanotubes [8], filopodia [32] reviewed in [1]). Considering this broad range of potential interactions, it is evident that monitoring cell-cell transmission, precise quantification of the events, and assessment of inhibitor efficacy has remained most complex. In part conflicting results obtained on neutralizing antibody efficacy in blocking HIV cell-cell transmission [13], [22], [23], [24], [25], [26] may be a consequence of the variable types of cell-cell interactions engaged in contacts between cells of different origin as well as differential assay set ups and readouts.

The primary intent of our current study was to derive a definite conclusion on the capacity of neutralizing antibodies to block cell-cell transmission of HIV. Our current knowledge of the mechanism by which antibodies neutralize HIV is largely based on data derived in assay formats which assess cell-free virus infection of a variety of target cells, either in single round or multiple round infection assays [33], [34], [35], [36], [37], [38]. While the former assays only monitor free virus entry, the latter measure a composite of free virus and cell-cell transmission during consecutive rounds of replication.

Several types of experimental approaches have been employed to dissect cell-free from cell-cell transmission when infected cells are used as source of virus inoculum. Single virus tracking by confocal microscopy [39], [40], disturbance of cell-cell contacts by keeping cultures in motion [14] and careful time course analysis of virus transmission to restrict analysis to a time window when mostly cell-cell transmission occurs [18], [22], [26]. The latter approach has been the most promising to date. Yet these assays require careful fine tuning of a relatively short infection interval. Virus transfer due to the short interaction can be relatively low, require sensitive detection systems and can be error prone [22]. Here we made use of assay systems which allow overcoming several limitations and explicitly monitoring cell-cell transmission. The comprehensive *in vitro* analysis of inhibitor activity during cell-free and cell-cell virus transmission that we present here provides a necessary first step towards the definition of the *in vivo* relevance of the respective transmission modes and ensuing requirements for their inhibition by vaccine induced antibody responses and entry inhibitors.

## Results

### Quantitative dissection of cell-cell and cell-free transmission of HIV-1

An inherent difficulty in dissecting neutralizing antibody action on cell-free and cell-associated virus is related with the respective assay systems used to evaluate the cell-cell transmission events. While cell-free infection can easily and most precisely be quantified (eg by using single-round infecting viruses), genuine cell-cell transmission is difficult to assess when transmission from infected to uninfected cells is studied. Replication competent virus is required in these settings. Although close cell contacts favor synapse formation and cell-cell transmission [14], entirely excluding the contribution of free virus transmission has thus far remained difficult.

**Figure 1:**
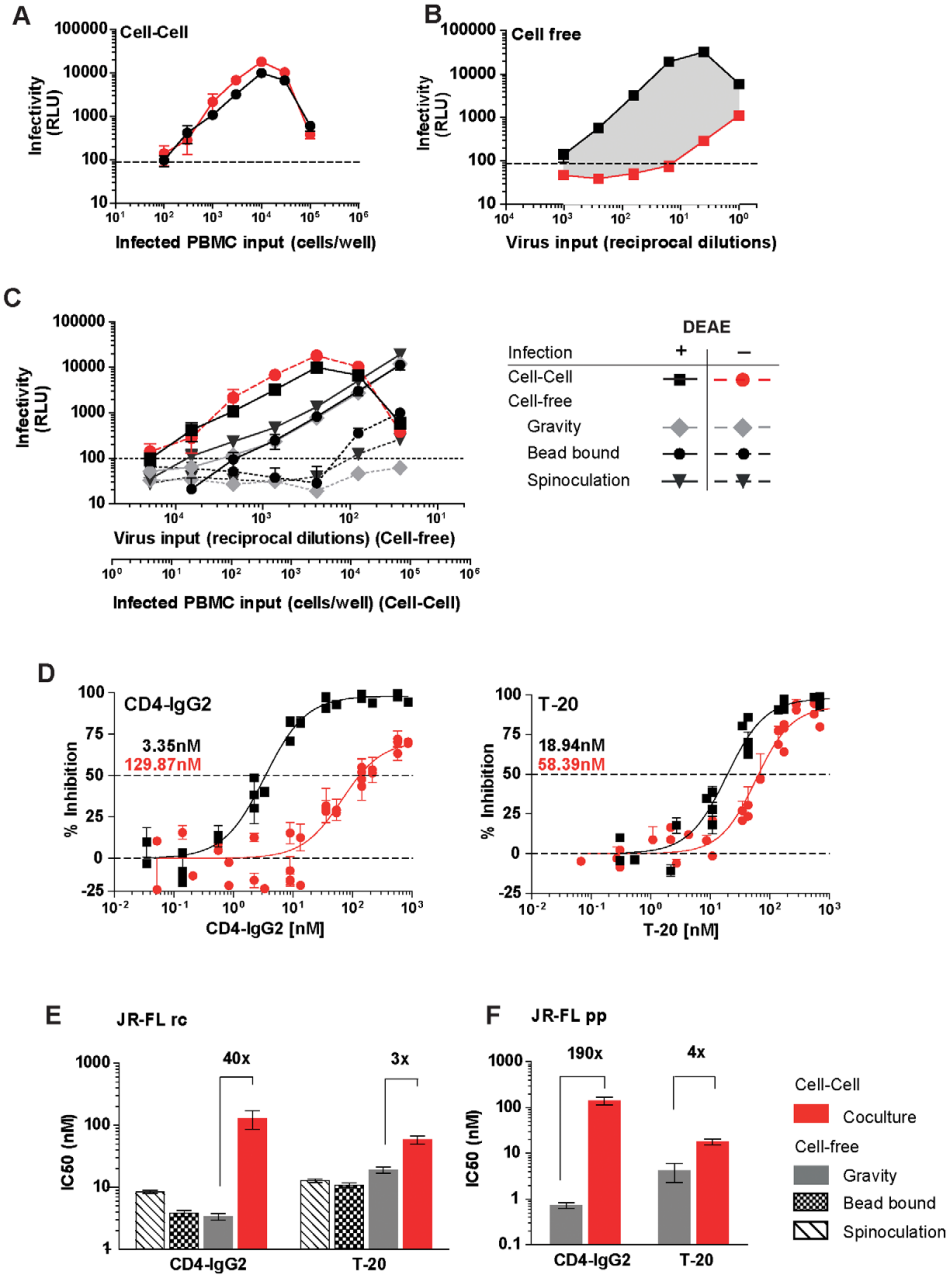
Mode of virus transmission differentially steers susceptibility to entry inhibition. **(A) DEAE-Dextran is not required for effective cell-cell transmission of HIV-1_JR-FL_ to TZM-bl cells.** Serial dilutions of JR-FL infected PBMC were incubated with TZM-bl cells in presence (black circles) or absence (red circles) of 10 µg/ml DEAE-Dextran. Infectivity was measured by enzymatic activity of the luciferase reporter (relative light units (RLU)). Each infected cell input was probed in triplicate. Error bars represent SD (standard deviation). One of four independent experiments is shown. **(B) Omission of DEAE-Dextran as media supplement abolishes cell-free JR-FL infection of TZM-bl cells.** Serial dilutions of cell-free JR-FL virus were used to infect the luciferase reporter cell line TZM-bl in presence (black squares) or absence (red squares) of 10 µg/ml DEAE-Dextran. Infectivity was measured by induction of the luciferase reporter (relative light units (RLU)). Each virus dilution was probed in quadruplicates. Bars represent SD . One of four independent experiments is shown. **(C) Cell-cell transmission but not enforced contact between virus and target cell overcomes entry restriction.** The infectivity of cell-free virus without enforced attachment to TZM-bl target cells (gravity sedimentation), or upon spinoculation, magnetic bead virus adsorption and during cell-cell transmission was assessed in presence (solid lines) or absence (dotted lines) of 10 µg/ml DEAE-Dextran. Infection was determined by measuring luciferase production after 48 h (recorded as RLU). Each virus dilution was probed in duplicates. Bars represent SD. One of three independent experiments is shown. **(D) Inhibitory profiles of CD4-IgG2 and T-20 during cell-cell and cell-free transmission.** TZM-bl target cells were either cocultivated with JR-FL infected PBMC (red circles, no DEAE) or cell-free virus (black squares, with 10 µg/ml DEAE) in the presence of increasing doses of CD4-IgG2 (left panel) or T-20 (right panel). Infection was determined by measuring luciferase production after 48 h and recorded as RLU. Red and black values denote IC50 (nM) of during cell-cell and cell-free transmission, respectively. Data points represent means of duplicates from three independent inhibition experiments. Bars represent SEM. Lines depict fitted dose response curves. **(E) Decreased CD4-IgG2 sensitivity during cell-cell transmission is due to an inherent feature of cell-cell transmission.** TZM-bl target cells were mixed with replication competent infected JR-FL^rc^ PBMC in the presence of CD4-IgG2 or T-20 (red bars) in medium lacking DEAE Dextran. Cell-free JR-FL^rc^ was either spinoculated (hatched bars), adsorbed by magnetic beads (checkered bars) or added without enforced adsorption (grey bars) onto TZM-bl target cells in medium containing DEAE Dextran in the presence of the inhibitor. Fold increases in IC50 of cell-cell compared to cell-free infection are indicated on top of the respective bars. Bars depict means of three independent experiments in duplicates. Lines denote SD. Inhibition of cell-cell transmission by CD4-IgG2 and T-20 (red bars) was significantly less efficient than blocking of cell-free virus (grey bars) infection (Student t-test, p<0.0001 in both cases). **(F) Single round infection is highly resistant to CD4-IgG2 inhibition during cell-cell transmission.** TZM-bl target cells (no DEAE) were co-cultivated with JR-FL pseudovirus transfected 293-T cells in the presence of CD4-IgG2 or T-20. Cell-free JR-FL^pp-lucAM^ was added to the TZM-bl (with 10 µg/ml DEAE) in the presence of both inhibitors. Fold increases in IC50 of cell-cell compared to cell-free infection are indicated on top of the respective bars. Bars depict means of three independent experiments performed in duplicates. Lines denote SD. Inhibition of cell-cell transmission by CD4-IgG2 and T-20 (red bars) was significantly less efficient than blocking of cell-free virus (grey bars) infection (Student t-test, p<0.0001 in both cases).

To construct a robust high-throughput system allowing direct comparisons between cell-free and cell-cell transmission we chose the widely used luciferase reporter cell line TZM-bl as target cells [34]. PBMC infected with primary, replication competent (rc) virus isolates served as donor cells in our cell-cell transmission system as these should most closely resemble *in vivo* infected cells. Direct co-culturing of infected PBMC (PBMC^HIV+^) with TZM-bl cells results in rapid and efficient infection of these cells and can be monitored by induction of the reporter luciferase (Fig. 1A). In order to adapt the PBMC^HIV+^/TZM-bl infection system to specifically quantify cell-cell transmission we made use of the fact that many CCR5 (R5) using HIV strains are only capable of efficiently infecting engineered, CCR5 and CD4 expressing target cell lines such as TZM-bl in the presence of polycations [34], [41], [42]. We found that whilst cell-free infection by the R5 isolate JR-FL was dramatically reduced by the omission of DEAE Dextran (Diethylaminoethyl Dextran) (Fig. 1B), cell-cell transmission between JR-FL infected PBMC and the TZM-bl target cells was polycation independent (Fig. 1A). Free virus released from infected cells in the cell-cell transmission set up failed to infect in absence of polycation (Fig. S1A).

In line with previous reports [9], [11], [12], [13], [14], [22], HIV infection kinetics in the PBMC^HIV+^/TZM-bl transmission assay were accelerated compared to free virus infection (Fig. S1B). Of note, regardless of whether cell-free virus adsorption was enforced by spinoculation [43] or magnetic beads [44], entry of cell-free HIV-1_JR-FL_ into TZM-bl cells remained severely restricted when no polycation was added (Fig. 1C), reinforcing the notion that enhanced virus transmission during cell-cell contact involves activities that extend beyond a mere increase in membrane proximity. In order to discriminate cell-cell from cell-free virus transmission in the PBMC^HIV+^/TZM-bl infection assay, polycation dependent virus isolates were used (Fig. S2). Input of infected donor cells and cell-free virus input was calibrated so that infection of both occurs in the linear range of the assay system (Fig. 1A and 1B) and that free virus cannot infect in absence of DEAE-Dextran (Fig. 1B and S2A). In sum, these assay conditions allowed precise quantification of cell-cell transmission without interference of free virus infection in the PBMC^HIV+^/TZM-bl infection system.

### Mode of virus transmission differentially steers susceptibility to entry inhibitors

Using defined DEAE-Dextran dependent virus isolates (Fig. S2), we next employed the PBMC^HIV+^/TZM-bl cell-cell transmission assay to evaluate whether the mode of HIV transfer has an influence on the potency of neutralizing antibodies and entry inhibitors. We compared the inhibitory potency of the gp120-directed tetrameric CD4-IgG2 molecule (PRO 542) and the gp41-directed fusion inhibitor T-20 against the isolate JR-FL in cell-free and cell-cell virus transmission. Strikingly, inhibition of cell-cell transmission by CD4-IgG2 required an approximately 40-fold higher 50% inhibitory dose (IC50) than inhibition of the same virus strain during cell-free infection (Fig. 1D). In contrast, the gp41 directed fusion inhibitor T-20 was markedly less affected by the transmission mode requiring only 3-fold higher IC50 doses during cell-cell transmission. To verify whether the decreased sensitivity towards CD4-IgG2 during cell-cell transmission was merely due to more efficient adsorption of the virus to the target cells or an inherent feature of cell-cell transmission, we assessed the inhibitory capacity of CD4-IgG2 and T-20 against cell-free virus adsorbed to target cells by spinoculation or by magnetic beads (Fig. 1E). Both inhibitors remained equally active regardless whether adsorption of cell-free virus was increased or not, indicating that indeed cell-cell transmission associated events caused the loss of CD4-IgG2 activity rather than simple virus concentration on the target cell surface.

To restrict our assessment to the first round of cell-cell transmission events we next probed the efficacy of CD4-IgG2 and T-20 against cell-free JR-FL envelope pseudotyped virus (pseudovirus particle, pp) and in cell-cell transmission using 293-T cells transfected with plasmids encoding JR-FL pseudotyped virus as donor cells (Fig. 1F). Like replication competent virus, the JR-FL^pp^ proved more resistant to CD4-IgG2 inhibition during cell-cell transmission and required 190-fold higher concentrations to achieve 50% inhibition than cell-free pseudovirus (Fig. 1F).

### Gp120 specific entry inhibitors have decreased capacity to block cell-cell transmission

**Figure 2:**
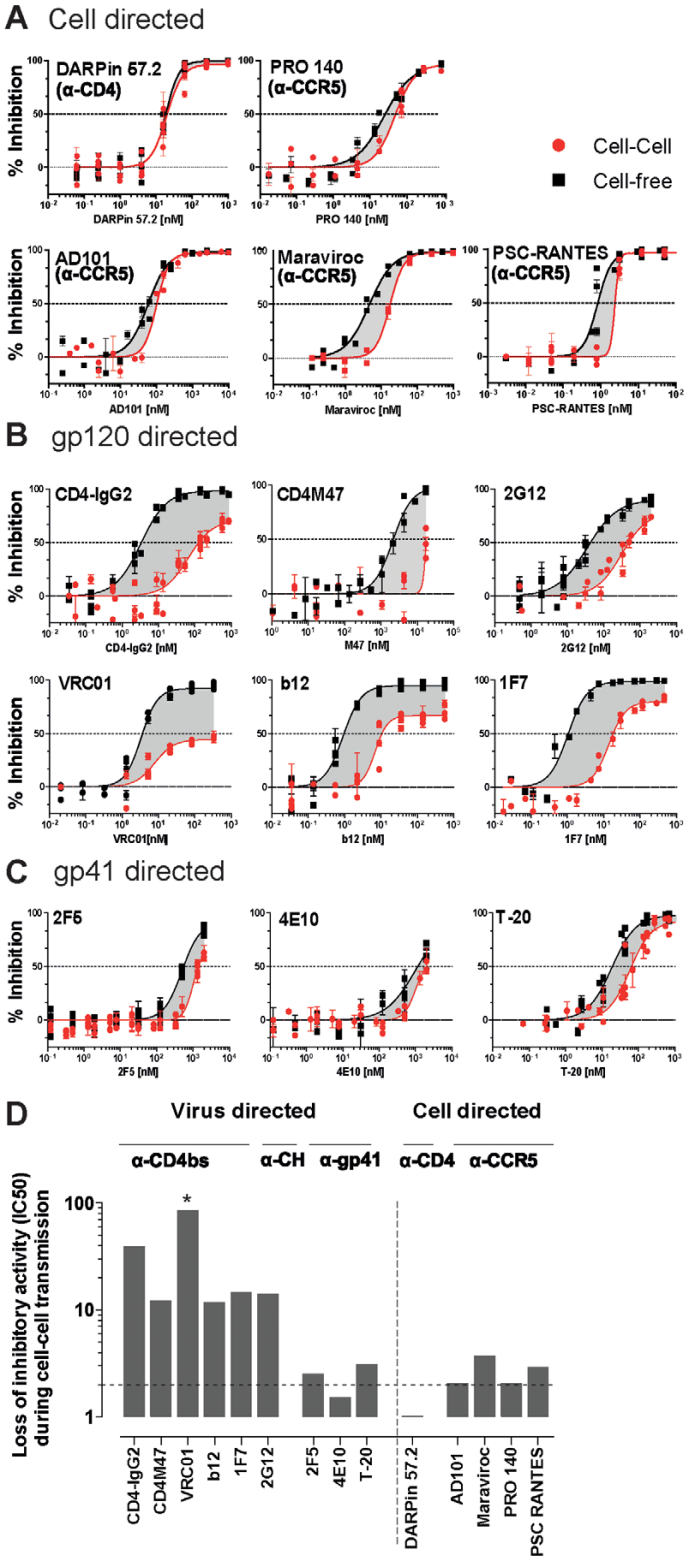
Markedly decreased sensitivity of HIV entry to gp120 directed inhibitors during cell-cell transmission. (**A–C**) TZM-bl target cells were either infected with cell-free JR-FL^rc^ (black squares, with DEAE) or cocultivated with JR-FL^rc^ infected PBMC (red circles; no DEAE) and inhibition by cell directed (A), gp120 directed (B) and gp41 directed (C) antibodies and inhibitors studied. Infection was determined by measuring luciferase production after 48 h (recorded as RLU). Lines depict fitted results derived from three to five independent experiments in which each sample condition was performed in duplicates. Error bars depict SEM. Dotted lines indicate 50% inhibition levels. **(D) Loss of inhibitory activity during cell-cell transmission.** Loss of inhibitory activity during cell-cell transmission compared to cell-free transmission is depicted as fold difference of IC50 values determined from data depicted in Fig. 2A–C. A star (*) denotes where the respective inhibitor did not reach a 50% inhibition level at the highest concentration used. The highest concentration probed was used in these cases as minimum estimate to derive the fold differences in IC50 values.

Our initial observations of the distinct effect of cell-cell transmission on CD4-IgG2 and T-20 activity raised the question whether the epitope specificity or neutralization mechanism of a given inhibitor determines its activity during cell-cell transmission of HIV. To probe this we investigated a panel of well characterized neutralizing antibodies and entry inhibitors for their respective potencies against cell-free and cell-associated virus. We selected inhibitors based upon their mode of action: cell- directed (CD4 or coreceptor CCR5 blocking; Fig. 2A), virus directed (gp120 (Fig. 2B) and gp41 specific (Fig. 2C)). Whenever possible inhibitors that differ in molecular mass and chemical structure (peptide, small molecule inhibitor, and antibody) were included for comparison (Table S1).

Comparison of the inhibitor activity under the two transmission modes revealed an intriguing pattern (Fig. 2D). While cell-directed inhibitors (anti-CD4, anti-CCR5) blocked cell-cell and cell-free transmission of JR-FL with almost identical efficacy (Fig. 2A and D, <4-fold loss of activity), HIV-1 envelope directed inhibitors showed a remarkably dichotomous pattern (Fig. 2B–D). All CD4 binding site specific agents (mAbs b12, VRC01, 1F7, the tetrameric CD4-IgG2 molecule and the CD4 mimetic CD4M47 [45]) lost considerable potency when cell-cell transmission occurred (10 to 100 fold decrease in activity reflected in according increase in IC50). Of particular note were the results we obtained for mAb VRC01. While VRC01 is one of the most potent antibodies in inhibiting cell-free transmission described to date [46], [47], it proved particularly ineffective in inhibiting cell-cell transmission of JR-FL. Similarly to the CD4bs specific agents, the carbohydrate specific mAb 2G12 also lost considerable activity when blocking of cell-cell transmission was required. This was in sharp contrast to the gp41 specific agents, the MPER-targeting neutralizing antibodies 2F5 and 4E10 and the fusion inhibitor T-20 which were all only marginally affected by the mode of virus transmission (Fig. 2C). Particularly surprising were the activities of the two MPER specific mAbs, despite the fact that they are not potent inhibitors of cell-free JR-FL virus transmission, their ability to block cell-cell transmission remained in the same range.

**Figure 3:**
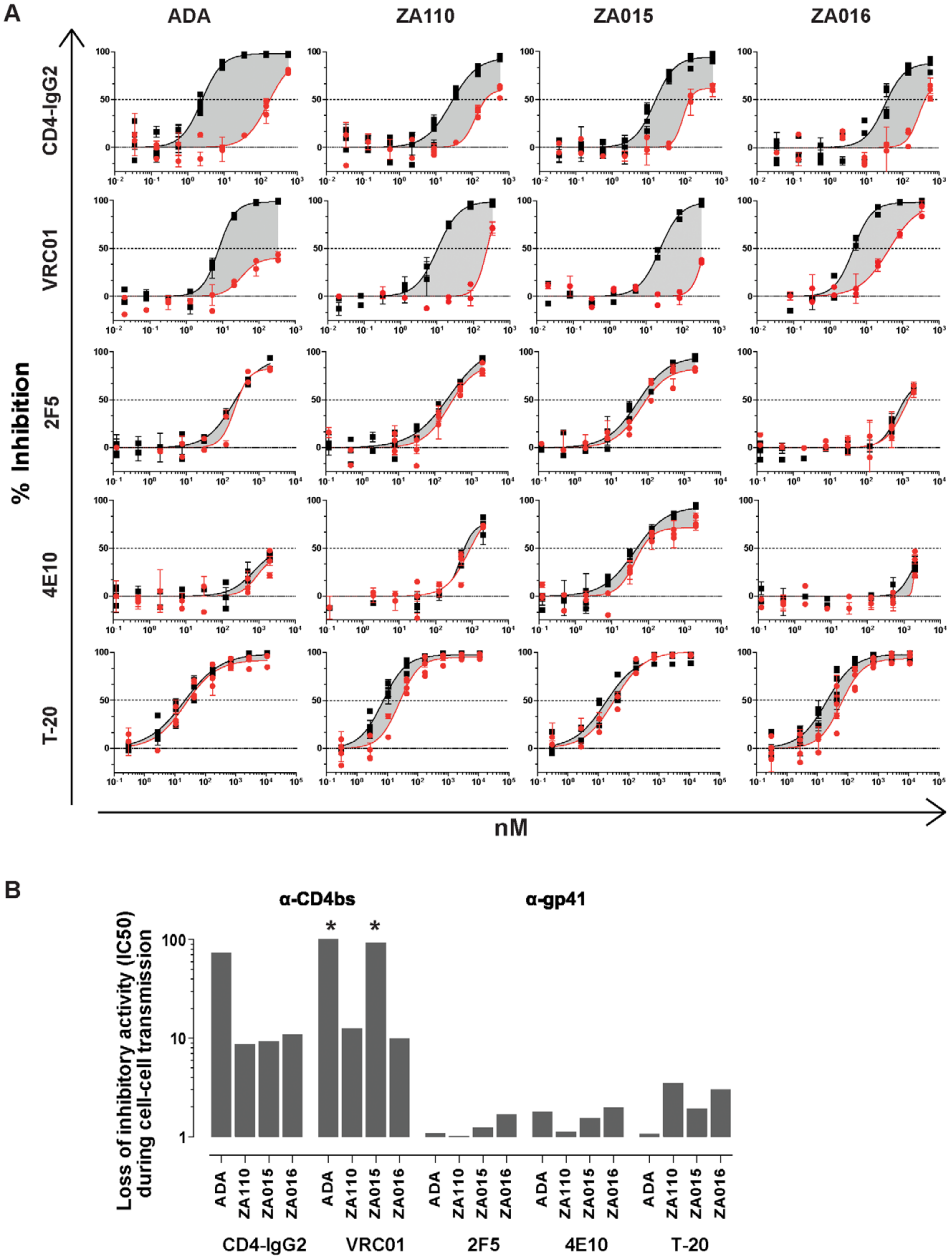
CD4bs directed inhibitors loose while gp41 directed agents maintain activity during cell-cell transmission across divergent HIV-1 isolates. (**A**) TZM-bl target cells were either infected with cell-free, replication competent viruses (black squares, with DEAE) or cocultivated with infected PBMC (red circles; no DEAE) and inhibition by the indicated antibodies and inhibitors studied. Virus isolates used (ADA, ZA110, ZA015 and ZA016) are indicated on top of the respective columns, inhibitors on the left of the respective rows. Infection was determined by measuring luciferase production after 48 h (recorded as RLU). Lines depict fitted results derived from two to three independent experiments in which each sample condition was probed in duplicates. Error bars depict SEM. Dotted lines indicate IC50 values. **(B) Loss of inhibitory activity during cell-cell transmission.** Loss of inhibitory activity during cell-cell transmission compared to cell-free transmission is depicted as fold difference of IC50 values determined from data depicted in Fig. 3A. A star (*) denotes where the respective inhibitor did not reach a 50% inhibition level at the highest concentration used. The highest concentration probed was used in these cases as minimum estimate to derive the fold differences in IC50 values.

The data we obtained thus far supported the notion that virus directed entry inhibitors fall into two distinct classes with a differential activity during cell-cell transmission: such that lose potency (eg CD4bs directed agents) and such which appear largely unaffected in their activity irrespective of the virus transmission mode (gp41 directed agents). We next verified the differential activity of specific CD4bs directed agents (CD4-IgG2 and VRC01) and the gp41 directed agents (2F5, 4E10 and T-20) in cell-cell and free virus transmission using four genetically divergent viruses, the Tier-1 virus ADA, the Tier-2 isolates ZA015, ZA016 and the Tier-3 isolate ZA110 (Fig. 3). The same pattern of reactivities was also seen for these viruses: CD4bs directed agents lost substantial potency during cell-cell transmission, while MPER mAbs and T-20 were only marginally affected (<4-fold for MPER mAbs).

### Efficient inhibition of T-cell to T-cell transmission by gp41 directed inhibitors

While the PBMC^HIV+^/TZM-bl assay proved very robust and provides a means to study cell-cell transmission under tightly controlled conditions, it has two major limitations. For one, only R5 viruses which depend on polycation in order to infect the engineered target cells can be studied. Secondly, while TZM-bl cells are widely used as target cells in HIV neutralization assays, they are of epithelial origin and engineered to express CD4 and CCR5 in abundance [41]. Considering that type and densities of cellular receptors engaged in forming the virological synapse may differ to some extent depending on the types of cells engaged, we thought it prudent to verify our observations in a setting of T-cell to T-cell transmission.

To this end we employed an alternate assay system making use of the intracellular HIV restriction factor TRIM5α. While HIV has adapted to human TRIM5α (huTRIM5α), HIV infection is potently restricted by rhesus macaque TRIM5α (rhTRIM5α) which acts post-entry at steps preceding integration [48], [49], [50]. Notably this restriction appears to limit cell-free virus infections, but not cell-cell transmission [51]. We made use of this selective action of rhTRIM5α and generated A3.01-CCR5 T-cells which co-expressed GFP and either huTRIM5α or rhTRIM5α as described [51]. While the parental A3.01-CCR5 T-cells are permissive for HIV and cells transduced with huTRIM5α remained permissive, rhTRIM5α expression rendered the A3.01-CCR5 T-cells highly resistant to infection by cell-free virus (Fig. S3A) but not to infection by HIV via the cell-cell transmission route (Fig. S3B). Most importantly for our transmission studies rhTRIM5α restriction of cell-free infection occurs irrespective of coreceptors usage (Fig. S3A) and hence allows measurement of cell-associated virus transmission with a wider spectrum of virus isolates.

**Figure 4:**
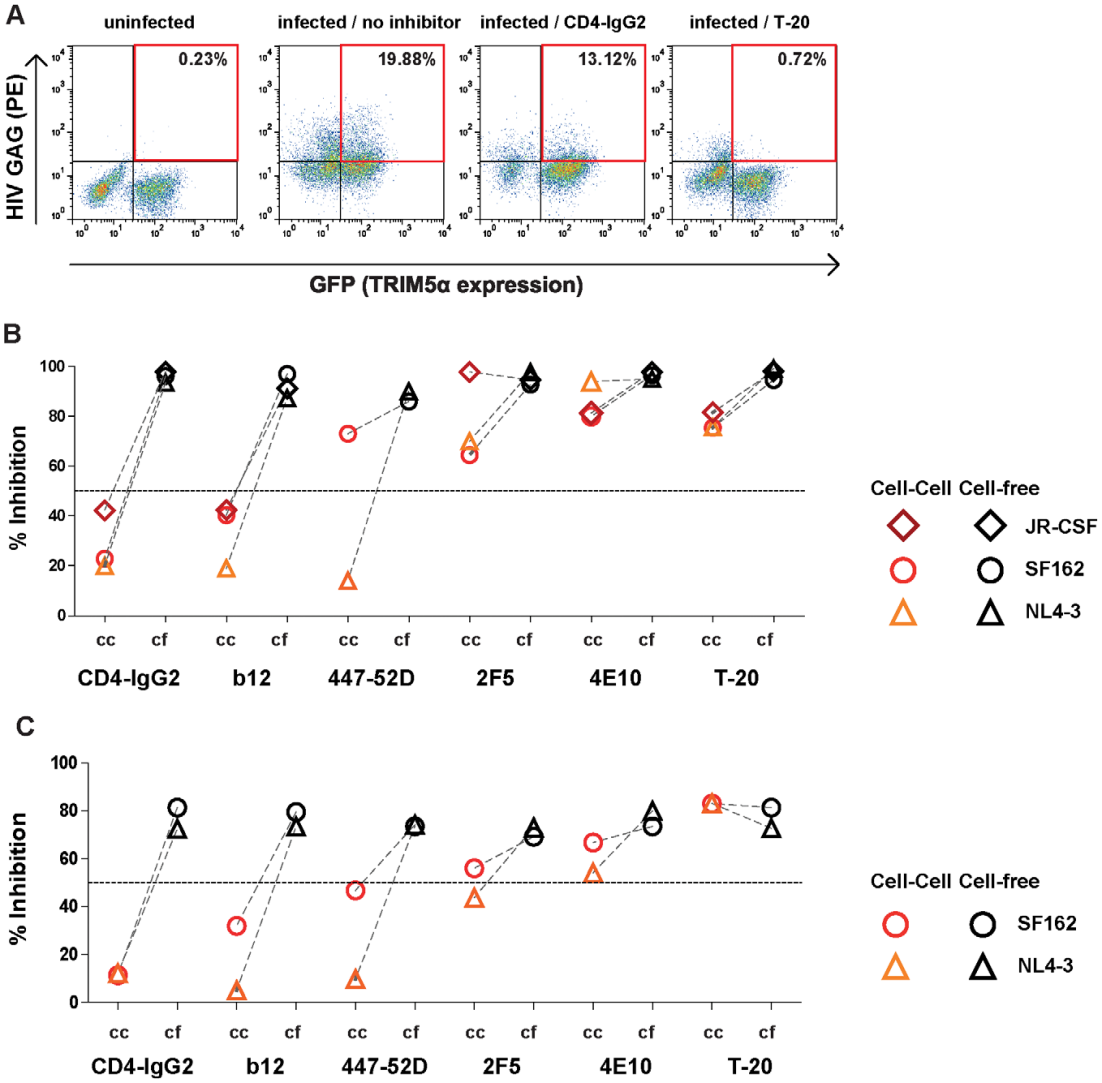
Efficient inhibition of T-cell to T-cell transmission by gp41 directed inhibitors. **(A) Inhibition of T-cell to T-cell transmission.** A3.01-CCR5 infected with JR-CSF or uninfected controls were co-cultured with A3.01-CCR5^rhTRIM5α^ target cells (GFP positive) in the presence of the indicated inhibitors or medium alone. Infection of target cells was assessed by intracellular Gag staining by flow cytometry. Percentages of rhTRIM5α expressing, HIV infected cells are indicated. One representative of two independent experiments is shown. **(B) Comparison of cell-free and cell-cell inhibition in rhTRIM5α restricted A3.01-CCR5 cells.** Inhibition of cell-cell (cc, red and orange symbols) and cell-free (cf, black symbols) transmission of virus isolates JR-CSF, SF162 and NL4-3 by inhibitors (CD4-IgG2, b12 and 447-52D: 50 µg/ml, 2F5, 4E10: 100 µg/ml, T-20: 5 µg/ml, see also Table S2) was studied. To probe cell-cell transmission infected A3.01-CCR5 were cocultured with A3.01-CCR5^rhTRIM5α^ target cells. To study free virus transmission cell-free virus preparations were used to infect non-restricted A3.01-CCR5 cells. Infection of target cells was assessed by intracellular Gag staining by flow cytometry as described in A). Infection achieved in absence of inhibitor was set to 100% and inhibitor activity expressed in relation to this value. Data depicted are means of two to seven independent experiments. **(C) Comparison of cell-free and cell-cell inhibition in rhTRIM5α restricted PBMC.** Inhibition of cell-cell (cc, red and orange symbols) and cell-free (cf, black symbols) transmission of virus isolates SF162 and NL4-3 by inhibitors (CD4-IgG2, VRC01, b12 and 447-52D: 50 µg/ml, 2F5, 4E10: 100 µg/ml, T-20: 5 µg/ml) was studied. To probe cell-cell transmission infected PBMC were cocultured with PBMC^rhTRIM5α^ target cells. To study free virus transmission cell-free virus preparations were used to infect non-restricted PBMC cells. Infection of target cells was assessed by intracellular Gag staining by flow cytometry as described in A). Infection achieved in absence of inhibitor was set to 100% and inhibitor activity expressed in relation to this value. Data depicted are means of two independent experiments in duplicates.

To probe the effect of entry inhibitors in T-cell to T-cell transmission we performed inhibition assays using HIV infected A3.01-CCR5 cells (A3.01-CCR5^HIV+^) as donor cells and rhTRIM5α expressing A3.01-CCR5 cells as targets (A3.01-CCR5^rhTRIM5α^) (Fig. 4A and B). We observed the same pattern of virus specific entry inhibition as in the PBMC^HIV+^/TZM-bl assay (Fig. 2). Gp41 directed inhibitors had similar or only slightly reduced activities in inhibiting the Tier-1 viruses SF162 (R5) and NL4-3 (X4) and the Tier-2 isolate JR-CSF (R5) during cell-cell transmission. In contrast CD4bs directed agents lost again considerable potency during cell-cell transmission. The V3 loop specific neutralizing antibody 447-52D [52], showed a strain dependent pattern. While 447-52D inhibition of NL4-3 was decreased from 90% to 14% during cell-cell transmission, only a marginal loss of SF162 inhibition occurred. Cell-free NL4-3 and SF162 is inhibited by 447-52D with similar potency [53], suggesting that differential V3 loop exposure during the entry process steers the efficacy of the mAb during cell-cell transmission, rather than higher potency. In line with this a second V3 loop antibody 1-79 [54] also blocked cell-free and cell-cell transmission of SF162 with identical potency (Fig. S4A).

To verify our findings in a setting where transmission was studied solely on primary T-cells, we generated rhTRIM5α expressing PBMC and monitored their infection by cell-free virus and cell-associated virus using HIV infected PBMC (Figure 4C). The data obtained in the PBMC^HIV+^/PBMC^rhTRIM5α^ assay confirmed our findings in the PBMC^HIV+^/TZM-bl and A3.01-CCR5^HIV+^/A3.01-CCR5^rhTRIM5α^ assays, and showed decreased activity of CD4bs antibodies, strain dependent reduction of V3 mAb inhibition and comparable activity of gp41 directed inhibitors during cell-cell transmission.

### Capacity to interfere with HIV attachment to target cells is not a prerequisite for neutralizing antibodies to block cell-cell transmission

**Figure 5 ppat-1002634-g005:**
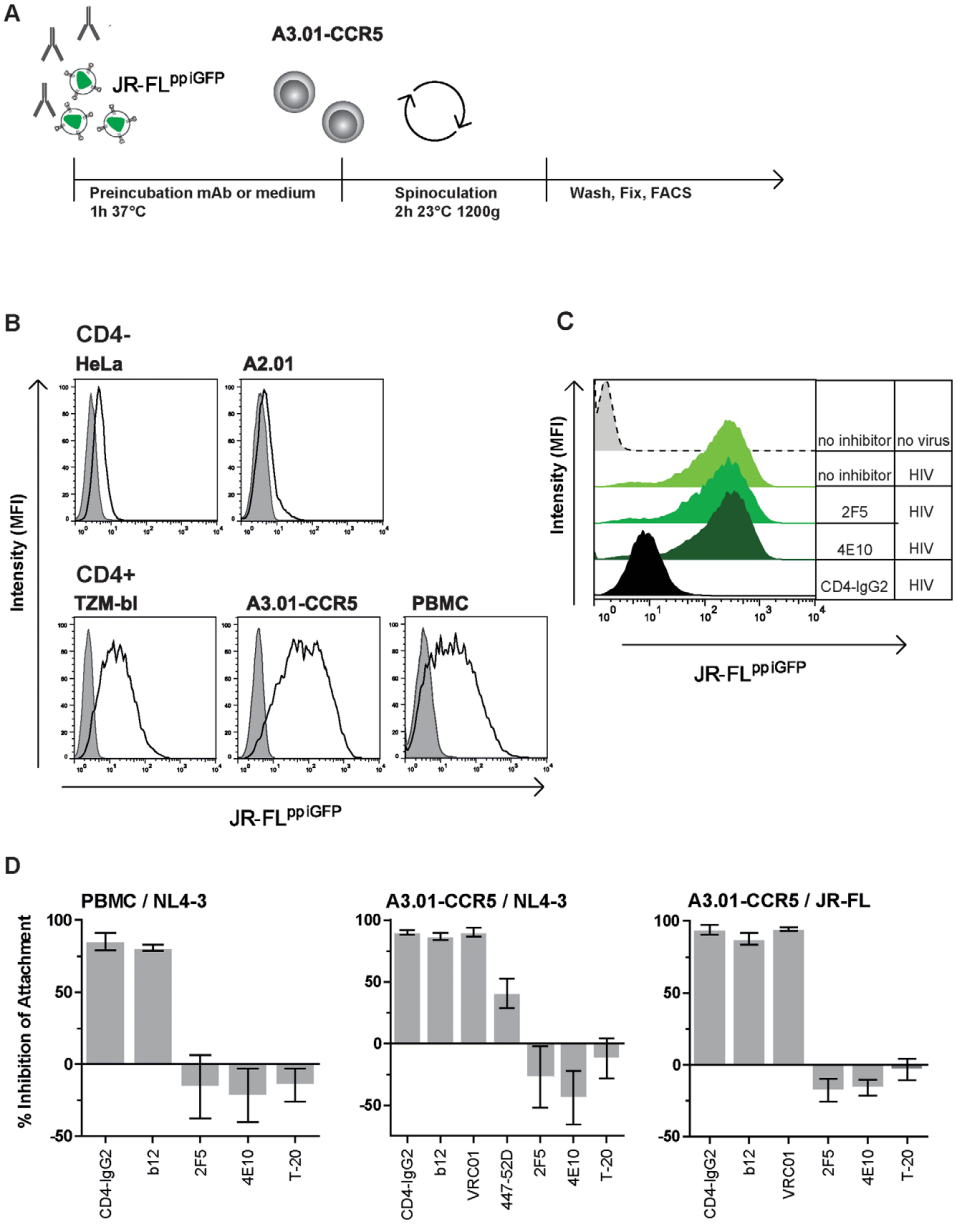
Attachment of virus is blocked by preventing gp120-CD4 interaction. (**A**) Schematic illustration of the experimental set up used to analyze virus attachment. **(B) Attachment of virus is driven by binding to CD4.** Attachment of HIV to CD4 negative (HeLa, A2.01) and related CD4 positive cells (TZM-bl, A3.01-CCR5) as well as stimulated, CD8 depleted PBMC was studied using GFP-labeled virus (JR-FL^ppiGFP^). The gray-shaded areas represent the fluorescent signal obtained by flow cytometric analysis of the respective cell line in the absence of HIV. The black lines indicate fluorescence intensity of bound JR-FL^ppiGFP^. **(C) Influence of entry inhibitors on HIV attachment.** Activity of 2F5 (100 µg/ml), 4E10 (100 µg/ml) and CD4-IgG2 (50 µg/ml) to block attachment of GFP-labeled virus (JR-FL^ppiGFP^) to A3.01-CCR5 cells is shown. Histograms of one representative of three independently performed experiments are shown. **(D) Inhibition of HIV attachment by CD4bs and gp41 directed agents.** Attachment (MFI of GFP signal) achieved in absence of inhibitor was set to 100% and inhibitor activity expressed in relation to this value. Data depicted are means of three independent experiments, error bars denote SEM. Left panel: Attachment of Vpr-GFP labeled TN8 virus (NL4-3 envelope) to PBMC. Middle panel: Attachment of GFP-labeled virus (JR-FL^ppiGFP^) to A3.01-CCR5. Right panel: Attachment of GFP-labeled virus (NL4-3^ppiGFP^) to A3.01-CCR5 cells (individual inhibitor concentration are listed in Table S2).

Our analysis of entry inhibitor activity in cell-cell transmission thus far had revealed a dichotomous pattern for virus envelope directed agents. While most gp120 directed agents, and in particular CD4bs agents, suffered from a considerable loss in activity during cell-cell transmission, gp41 directed inhibitors maintained their activity. We hypothesized that the basis for this dichotomy could be a genuine difference in inhibition modes and that the capacity to inhibit a specific phase of the entry process determines efficacy in blocking cell-cell transmission. To explore this we first evaluated the capacity of neutralizing antibodies to block attachment of fluorescently labeled HIV to target cells during spinoculation (Fig. 5A). Within this setup virus binding to a variety of cell lines and PBMC proved to be predominantly driven by binding of virions to CD4 (Fig. 5B). In contrast to previous reports [55], only marginal attachment of HIV to CD4 negative cells was detected in our assay set up. CD4 independent attachment of HIV to target cells was previously found predominantly amongst X4 isolates which were shown to bind cell surface expressed glycosaminoglycans (GAG) when target cells were incubated with concentrated virus stocks at 37°C [55], [56]. Our current analysis required assessment of attachment in a setting where binding of virions to cells is both, synchronized and entry halted before fusion. We achieved this by using spinoculation and a temperature arrest at 23°C and found that under these conditions non-CD4 driven attachment is negligible.

In line with the CD4 dependence in the attachment assay, CD4bs directed inhibitors, b12, VRC01 and CD4-IgG2 potently inhibited binding of JR-FL (R5) and NL4-3 (X4) to target cells (Fig. 5C and 5D). Interestingly, the V3 loop specific mAb 447-52D possesses a partial activity in inhibiting attachment of NL4-3 to target cells, suggesting that in some virus/antibody pairings co-receptor engagement may play a role in establishing firm attachment. In contrast MPER-directed antibodies 2F5 and 4E10 and the fusion inhibitor T-20 were not able to inhibit attachment. The latter is in accordance with the previously described limited capacity of MPER mAbs to neutralize virions before CD4 engagement [38], [57], [58].

### Neutralizing antibodies with post-attachment activity maintain potency during cell-cell transmission

**Figure 6 ppat-1002634-g006:**
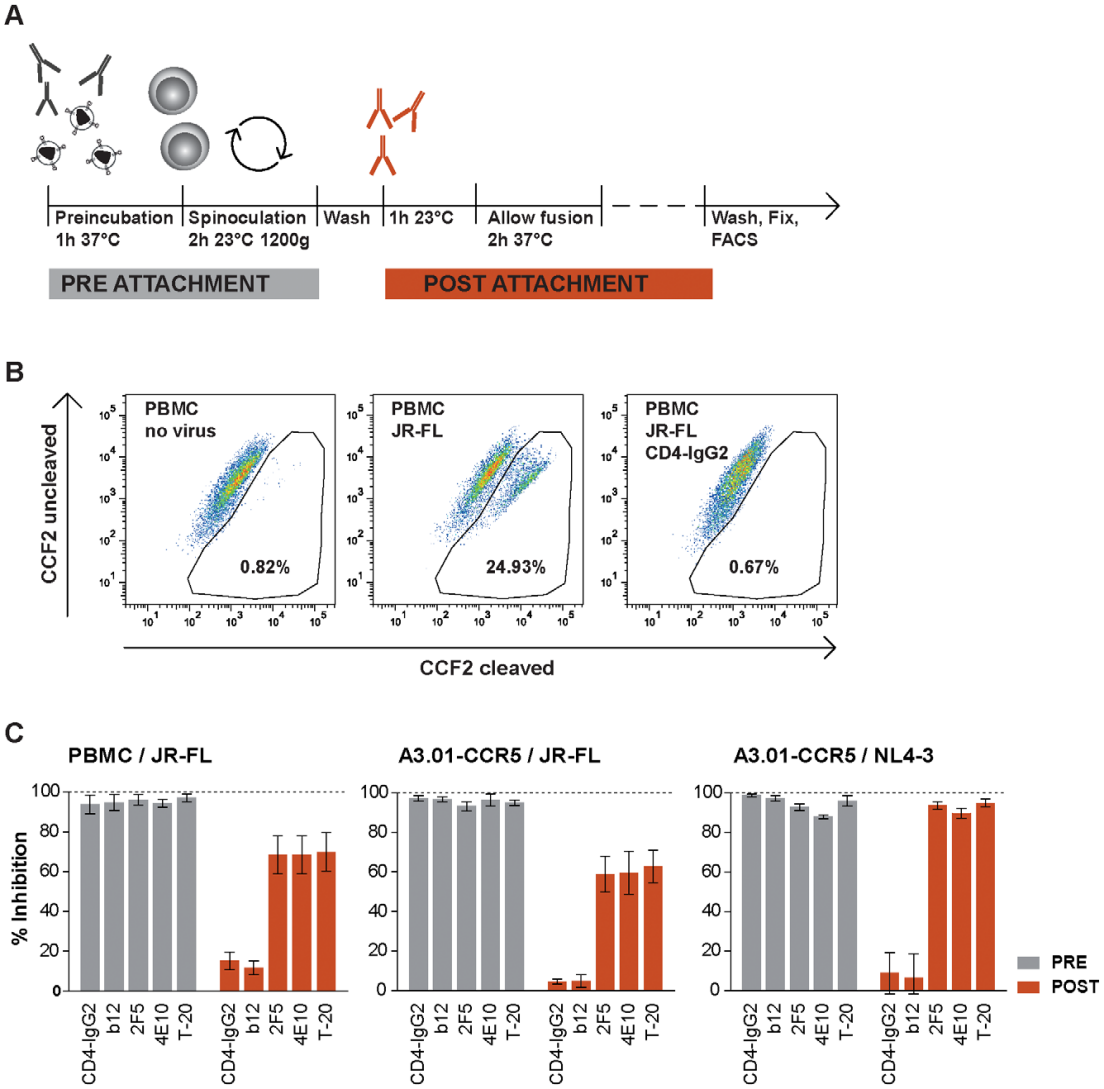
Post attachment activity of entry inhibitors. (**A**) Schematic illustration of the entry assay using BlaM-Vpr labeled virions. (**B**) JR-FL^pp-BlaM^ entry into PBMC was studied in presence and absence of CD4-IgG2 (50 µg/ml). One of three individual experiments is shown. Fluorescence of uncleaved CCF2/AM was recorded at 520 nm, β-lactamase cleaved CCF2/AM denoting HIV entry at 447 nm. **(C) Inhibition of virus entry.** Fusion of JR-FL^pp-BlaM^ and NL4-3^rc-BlaM^ with PBMC or A3.01-CCR5 cells was monitored in presence and absence of the indicated entry inhibitors (Inhibitor concentration: CD4-IgG2 and b12: 50 µg/ml, 2F5 and 4E10 : 100 µg/ml, T-20 10 µg/ml). Grey and orange bars correspond to pre- and post-attachment conditions respectively as depicted in (A). Data shown are means of three independent experiments, error bars denote SEM.

Following gp120 binding to CD4, HIV-1 enters its target cell in a multistep, temporally defined process (reviewed in [59]). In order to measure the inhibitory capacity of neutralizing antibodies on virus entry at two different stages of the infection process, we assessed virus-cell fusion utilizing β-lactamase (BlaM) loaded virions as described [60], [61] (Fig. 6A). Inhibitors were either added before HIV attachment to target cells and were removed following spinoculation or alternatively added after HIV attachment to target cells, hence providing a method by which post-CD4 engagement inhibitory activity can be measured. As expected when inhibitors were added before virion binding to PBMC or A3.01-CCR5 target cells and were present throughout the attachment process, all probed compounds potently blocked virus fusion (Fig. 6B and C). However, addition of inhibitors after attachment of the virus to the target cells, revealed a dichotomous pattern. CD4bs reactive agents completely lost their activity while the MPER-specific antibodies and the gp41-directed fusion inhibitor T-20 still possessed substantial inhibitory activity. The capacity of the gp41-directed agents to block infection post CD4 recruitment, is in line with previous reports observing MPER mAbs and HR1 and HR2 targeting inhibitors which act at a prefusion stage [57], [58], [62], [63], [64], [65], [66].

This divergent pattern of reactivities of gp120 CD4bs and gp41 directed agents in the fusion assay paralleled their capacity for inhibition of cell-cell transmission, raising the possibility that neutralizing activity post-CD4 engagement is required for efficient blocking of cell-cell transmission.

**Figure 7:**
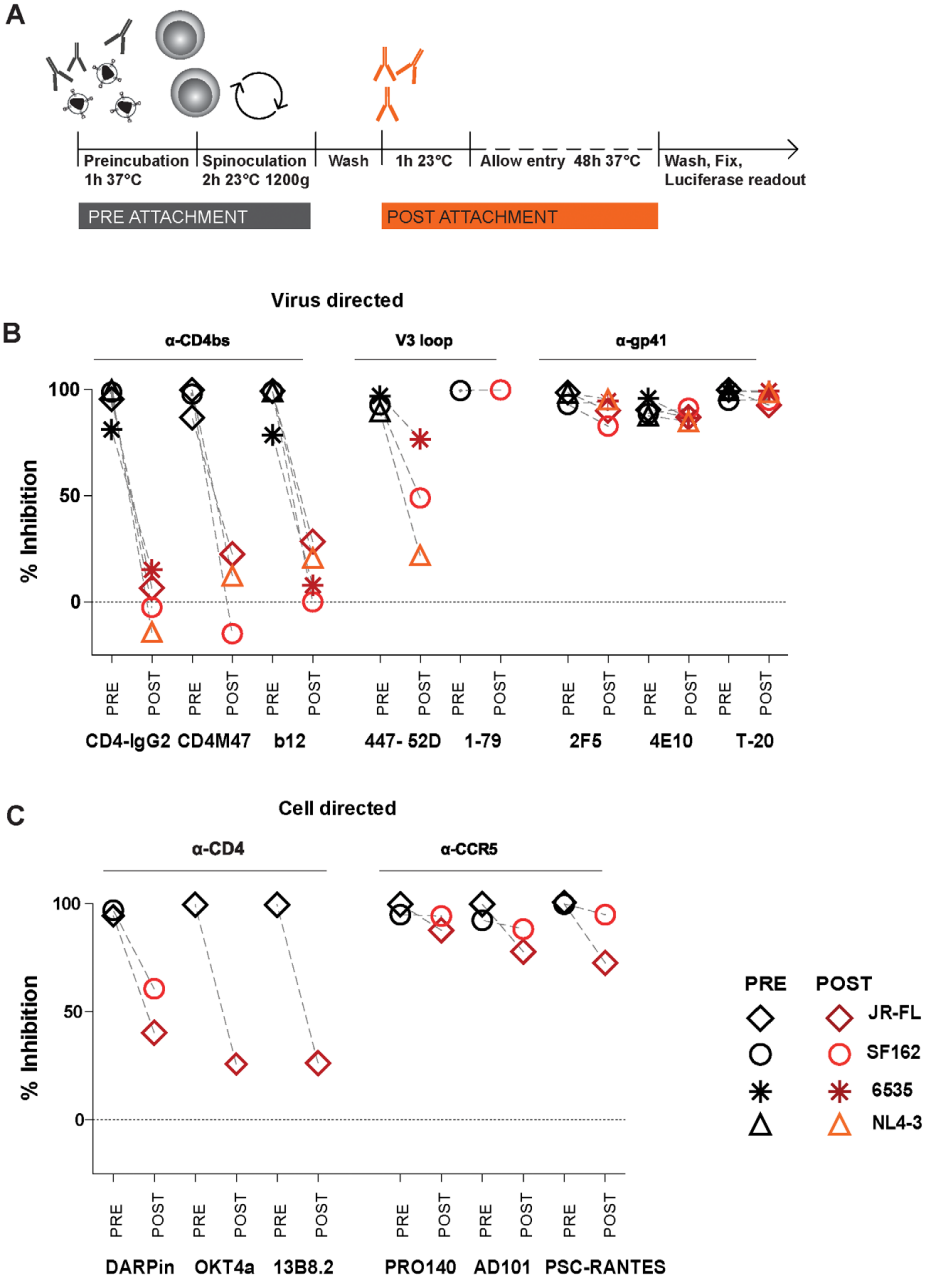
Gp41 specific inhibitors have broad post-attachment activity. (**A**) Schematic illustration of the luciferase reporter assay utilized to assess post attachment activity of inhibitors. **(B) Post-attachment activity of virus directed inhibitors.** A3.01-CCR5 cells were infected and treated with inhibitors before or after virus attachment as indicated in (A). (Inhibitor concentration listed in Table S2). Infection of env-pseudotyped, luciferase reporter viruses JR-FL^pp-lucAM^ (diamonds), SF162^pp-lucAM^ (circle), 6535^pp-lucAM^ (star), NL4-3^pp-lucAM^ (triangle) was determined after 48 h of culture by measuring luciferase production (recorded as RLU). Data depict means of pre attachment (black symbols) and post attachment activity (red and orange symbols) as % inhibition compared to untreated control. Means of three to six independent experiments are shown. **(C) Post-attachment activity of cell directed inhibitors.** A3.01-CCR5 cells were infected and treated with inhibitors before or after virus attachment as indicated in (A). (Inhibitor concentration listed in Table S2). Infection of env-pseudotyped, luciferase reporter viruses JR-FL^pp-lucAM^ (diamonds) and SF162^pp-lucAM^ (circle)was determined after 48 h of culture by measuring luciferase production (recorded as RLU). Data depict means of pre attachment (black symbols) and post attachment activity (red and orange symbols) as % inhibition compared to untreated control. Means of two to four independent experiments are shown.

To resolve pre- and post-attachment activity of inhibitors in more detail, we infected A3.01-CCR5 cells with envelope pseudotyped luciferase reporter viruses again performing treatment with neutralizing antibodies before or after attachment of the virus particles to the cells (Fig. 7A). For all four pseudoviruses probed (NL4-3, SF162, JR-FL and 6535) the potency of gp120 CD4bs directed reagents (CD4-IgG2, CD4M47 and mAb b12) was dramatically reduced when added after receptor engagement (Fig. 7B). Since the peptidic inhibitor CD4M47 experienced the same difficulties in blocking cell-cell transmission as the CD4bs antibody and CD4-IgG2 despite its small size, the limited capacity of mAbs to access the CD4bs during cell-cell transmission is unlikely to be responsible for their reduced activity during cell-cell transmission. In contrast to CD4bs agents, the potency of the gp41-directed inhibitor T-20 and the MPER-specific antibodies remained essentially unchanged when added after receptor engagement. The V3 loop mAb 447-52D showed an intermediate pattern, it lost more than 50% of its activity against NL4-3 and SF162, but activity against the isolate 6535 was preserved, indicating again that V3 loop exposure post CD4 binding varies in a strain dependent manner. Importantly, strain 6535 was inhibited with identical activity by 447-52D during cell-cell transmission (Fig. S4B). The same was true for the V3 loop mAb 1–79 which blocked SF162 potently post attachment (Fig.7C) and during cell-cell transmission (Fig. S4A).

Anti-CD4 directed agents (CD4-DARPin 57.2, anti-CD4 mAbs OKT4A [67] and 13B8.2 [68]) showed decreased activity when added post attachment, while anti-CCR5 inhibitors (AD101, PRO140, PSC-RANTES) had, in most cases, comparable activity when present before and after attachment (Fig. 7C). This observation suggests that under these assay conditions coreceptor engagement has not been fully established prior to inhibitor addition. The decreased activity of the anti-CD4 inhibitors in the post-attachment assay is expected from their mode of action. These CD4 receptor directed agents nevertheless block free-virus transmission and cell-cell transmission equally well, while CD4bs gp120 directed inhibitors do not. This highlights the advantage cell-directed inhibitors have, as their target is accessible before and during envelope attachment. In contrast virus-directed inhibitors only have a narrow window of opportunity to act - after virus envelope proteins are expressed and transported to the surface of infected cells.

## Discussion

The primary aim of our current study was to dissect the efficacy of neutralizing antibodies and entry inhibitors in the context of cell-cell transmission of HIV. It is generally agreed that neutralizing antibody responses will be a key component of an effective HIV vaccine [69], [70]). However, whether vaccine elicited neutralizing antibodies will need to block only the infecting inoculum, or whether protection will also require restriction of consecutive rounds of infection, and hence inhibition of both cell-free and cell-cell transmission with equal efficacy is not known.

Likewise, in established infection, should one of the transmission modes prove to be clearly dominant, this mode may need to be targeted preferentially by therapeutic vaccines and entry inhibitors.

While it is known that HIV spreads highly efficiently through various types of cell-cell contacts [9], [10], [11], [12], [13], [14], so far no consistent picture of antibody action during this process has emerged [13], [22], [23], [24], [25], [26]. Only few neutralizing antibodies and inhibitors have been probed for their efficacy in cell-cell transmission, amongst these are antibodies and inhibitors related to those used in our current study (b12 [71], 447-52D [52], 2F5 [72], 4E10 [73], [74]; anti-CD4 mAbs Leu3a and Q4120 [75], and anti-coreceptor inhibitors AMD3100 [76], TAK779 [77]). Several studies reported that MPER mAbs [13], CD4bs mAbs [13], [24], T-20 [13], and anti-coreceptor agents [4], [13] were significantly less potent inhibitors of cell-cell transmission than cell-free virus transmission. Others found cell-cell and cell-free neutralization activity to be equivalent (MPER mAbs [22], [25], [26], CD4bs mAbs [26], V3 mAb 447-52D [4], fusion inhibitors T-20 [22] and C34 [26], anti-CD4 agents [4], [22], [26], [40], and anti-coreceptor agents [22], [39]). However the wide range of assay systems used adds complexity to the interpretation and comparison of the results. Several experimental approaches did not allow direct comparison of cell-cell and cell-free transmission in the same setting [4], [13], [25], [26], [39], other assay systems do not allow precise quantification of inhibitory activity [4], [23], [25]. Additionally, all systems employed to study genuine cell-cell transmission thus far are technically challenging and can be error prone [22]. Nevertheless, in agreement with our observations, one study also reported lower activity of the CD4bs mAb b12 during cell-cell transmission, albeit this difference was rated as non significant by the authors [22].

Here we report on neutralizing antibody activity during cell-cell transmission using specifically tailored experimental strategies which enable unambiguous discrimination between the two transmission modes. The principle by which free virus infection can be distinguished from cell-cell transmission in these two systems is different. In the TZM-bl transmission assay free virus infection of specific R5 viruses can be restricted by omission of the polycation DEAE-Dextran in the infection medium (Fig. 1B and S2). In the second assay system we make use of the capacity of the restriction factor rhesus TRIM5α which potently interferes with free virus infection but not cell-cell transmitted virions (Fig. S3) [51]. While the mode and stage of free virus restriction in the two assay systems are different, the outcome of our analysis of neutralizing antibody capacity in both systems was identical. Gp120 directed inhibitors and neutralizing antibodies, in particular CD4bs directed agents, showed markedly decreased potency in blocking cell-cell transmission, whereas the probed gp41 directed inhibitors, the fusion inhibitor T-20 and the MPER antibodies 2F5 and 4E10, demonstrated identical or only marginally reduced potency. Of particular note are the cell directed inhibitors. Both CCR5 and CD4 targeting compounds were equally active during both transmission modes (Fig. 2A). Although they target the same step in virus entry, namely gp120 interaction with CD4, we found that anti-CD4 inhibition was not decreased in cell-cell transmission, while CD4bs mAb activity is markedly lower (Fig. 2B, 3 and 4BC). This is not unexpected. During cell-cell transmission, inhibitors targeting the cellular receptors have a clear advantage, CD4 and CCR5 receptors are always accessible on target cells and inhibitors can bind immediately. In contrast, virus directed inhibitors depend on the initiation of the viral synapse formation, as only then the viral envelope becomes accessible. As the viral synapse formation is tightly linked to gp120 binding to CD4 [3], [4], [13], CD4bs specific inhibitors are likely to only have a narrow time window for action as evidenced by their loss of activity during cell-cell transmission.

Only a limited number of entry inhibitors have thus far been probed for their activity during chronic infection in animal models, and even fewer have reached clinical investigation in therapeutic settings. At present only two inhibitors, the CCR5 specific inhibitor Maraviroc and the fusion inhibitor T-20 are in clinical use. Intriguingly, the currently available data may point towards a potential link between inhibitor action in established infection *in vivo* and activity during cell-cell transmission. The capacity of Maraviroc and T-20 to restrict cell-free and cell-cell transmission with equal efficacies, and the comparative failure of CD4-IgG2 to do the same, parallels their differential clinical success [78], [79], [80], [81], [82], [83]. It is tempting to speculate that potent inhibition of cell-cell transmission is a prerequisite for the therapeutic success of entry inhibitors during established infection. This would bode well for the development of cell-directed inhibitors as all CD4 and CCR5 directed inhibitors we probed potently blocked cell-cell transmission. Importantly this would also imply that cell-cell transmission is responsible for a substantial proportion of viral spread in infected individuals.

Will activity against free virus suffice for both prophylactic vaccine induced antibody responses and prophylactic interventions with entry inhibitors? Or is inhibition of cell-cell transmission also required in these settings? Judging from the success of animal protection/challenge studies performed with b12 [84], [85], a mAb which we find does not inhibit cell-cell transmission efficiently, one could speculate that potent activity against cell-cell transmission may not be necessary for prophylactic vaccines. Yet, a dual function of antibody based vaccines against both incoming cell-free virus and early cell-cell transmission could potentially enhance efficacy. Based on our observations we consider it thus prudent to incorporate cell-cell transmission studies in current pre-clinical and clinical vaccine assessment to determine whether or not activity in blocking cell-cell transmission is a correlate of protection.

It is very intriguing that amongst the neutralizing antibodies probed the activity of the MPER mAbs and T-20, were the least affected by the virus transmission modes. 2F5 and 4E10, in comparison, displayed only modest potency in free virus inhibition. Yet this activity was largely maintained during cell-cell transmission suggesting that the window of opportunity of action for these mAbs is similar during both entry processes. Several lines of evidence support this hypothesis. MPER specific antibodies can bind and neutralize free virions before receptor engagement [38], [86], however this process is slow, requiring several hours. In contrast these mAbs appear to act preferentially in a cellular context following HIV envelope engagement by CD4. Once the envelope trimer has bound cellular receptors and envelope rearrangements proceed, the MPER domain becomes more accessible allowing the antibodies to rapidly bind and neutralize the virus [57], [58], [86], [87], [88], [89]. In line with this we found that MPER mAbs were not able to inhibit attachment, but blocked fusion, both when added during or after attachment (Figs. 5–7). Thus MPER mAbs and T-20 can block virus that has already bound to receptors, highlighting that processes required for the transition from receptor engagement to fusion are slow enough for these agents to act. Most importantly in the context of our current study, this suggests that the timing of these processes is identical, regardless of whether free virus or cell-cell transmitted virus is concerned. Post-CD4 attachment activity was previously also reported for other neutralizing antibodies besides MPER mAbs and gp41 targeting inhibitors including V3 loop mAbs and small molecule inhibitors targeting CCR5 [64], [65], [66], [90], supporting our observations.

Inhibitors targeting the cellular receptor have immediate access to CD4 and the coreceptors both during cell-cell and cell-free transmission, corresponding to the identical activity we observed in both settings. In turn this highlights that the binding of gp120 to CD4 in the cell-cell transmission setting must be the rate limiting step for CD4bs directed agents. Virus specific antibodies and inhibitors, regardless of the epitope they recognize and their actual size, can only reach the virus envelope once it becomes exposed on the cell surface. When infected cells are in close proximity to potential target cells it is likely critical for neutralizing antibodies to reach the exposed envelope proteins in time, before they encounter target cell receptors and cell-cell transmission commences.

Once the trimer becomes accessible, CD4 engagement appears to be initiated too rapidly for CD4bs specific agents to block with equal efficacy as in cell-free virus transmission. We found that activity of other gp120 antibodies during cell-cell transmission such as 2G12 or V3 loop specific mAbs, which arrest virus infection post-CD4 engagement, can also be substantially reduced during cell-cell transmission. Interestingly for V3 loop specific antibodies we noted a differential activity during cell-cell transmission depending on the mAb and virus strain investigated (Figs. 4B, 4C and S4). How quickly a given virus envelope proceeds from CD4 engagement to coreceptor binding and fusion will likely be determined by its intrinsic reactivity [91] which may also influence activity during cell-cell transmission. The more rapid this process the less effective the respective antibody will likely be in blocking cell-transmission. In line with this we found that mAb 447-52D blocks virus strains NL4-3, SF162 and 6535 potently when present during attachment, but only strain 6535 at comparable levels when added post attachment. SF162 was also inhibited with identical potency when added during pre- and post attachment by the V3 loop mAb 1–79. Importantly in all cases we investigated, high post-attachment activity of V3 loop mAbs was associated with high efficacy in inhibiting cell-cell transmission (Figs. 4B, 4C, S4 and 7).

A key finding of our study is the failure of CD4bs specific antibodies to maintain their potency during cell-cell transmission. A wealth of data on this antibody category has emerged over recent years. CD4bs specific antibodies are ubiquitously elicited during natural infection [46], [92], [93], [94], [95], subject to escape [96], and undergo substantial somatic hypermutation to adapt [54], [94], [97], which can lead to the generation of broadly active, potent neutralizing CD4bs specific antibodies [46], [71], [94]. There is recent evidence that the evolution of potent neutralizing antibodies may follow similar paths across individuals and from different immunoglobulin heavy genes [94].

HIV escapes antibody responses rapidly [98], [99], [100], [101], [102]. Accordingly, even the most potent and broadly active antibodies characterized in recent years [46], [47], [94], [103], [104], [105], [106] have nonetheless been isolated from individuals who fail to control viremia. However due to their breadth and potency these mAbs may indeed prove to be the responses required for an effective, prophylactic vaccine. Nevertheless their failure to halt disease progression needs to be understood. Our observations may resolve the conundrum of how CD4bs mAbs can be so exorbitantly powerful *in vitro* and yet to our current knowledge lack comparable potency *in vivo* and fail to suppress viremia to undetectable levels for prolonged periods. We show here that the blocking activity of CD4bs antibodies is largely directed towards free virus, thereby restricting virus spread to the cell-cell route. In the resultant setting their blocking activity is vastly reduced, thus allowing virus replication and spread to occur. Simultaneously this partial inhibition scenario likely fosters escape as sufficient replication under a partial selection pressure is maintained. *In vitr*o and *in silico* studies of drug resistance evolution which factored in cell-cell transmission recently came to similar conclusions [18]. The continuous selection of virus escape variants, the high somatic hypermutation of CD4bs antibodies and the emergence of highly potent CD4bs directed neutralizing antibodies underline that these antibodies are continuously imposing a selection pressure on the virus.

In support of our findings, Poignard and colleagues previously observed that high serum concentrations of b12 and other neutralizing monoclonal antibodies, which provide protection against free virus challenge, lose their impact in an ongoing established infection in hu-PBL-SCID mice [107]. Of particular interest, b12 resistant virus rapidly emerged while wildtype, neutralization sensitive virus was maintained concurrently, a finding which corresponds to our proposed scenario where HIV may in part escape neutralization and maintain infection by cell-cell transmission.

Based on our observations it is tempting to speculate on the *in vivo* relevance of cell-free and cell-cell transmission. We hypothesize that the selection pressure provided by CD4bs mAbs should be stronger on free virus than on cell-cell transmitted virus. The fact that CD4bs antibodies can nevertheless maintain an apparently considerable and continuous selection pressure *in vivo* would argue in turn that free virus transmission must be an important component of viral spread in infected individuals. The importance of free virus transmission may thereby lie either in a quantitatively higher contribution to viral spread in the infected individual or a qualitative asset. Should virus spread preferentially occur through neutralizing antibody vulnerable free virus transmission, cell-cell transmission would allow the virus to maintain replication despite antibody pressure and foster rapid escape. Alternatively, should cell-cell transmission constitute a higher proportion of transmission events *in vivo*, we would argue that free virus transmission must nevertheless be important, otherwise the selection pressure on free virus transmission could not be so pronounced. It is likely that in the latter case cell-cell transmitted viruses still depend on free virus transmission to reach anatomically distant sites. This may also be crucial for dissemination of the virus in as target cell availability at the initial sites of replication will decrease. However, should cell-cell transmission indeed be the quantitatively dominant transmission mode, it is feasible that antibody responses which specifically restrict this transmission mode could emerge. With the panel of antibodies probed in our current study we saw preferential blocking of free virus not cell-cell transmission. It will be intriguing to probe larger antibody panels in future studies and to determine to what extent the recently defined, potent quaternary and carbohydrate specific mAbs [103], [104] inhibit cell-cell transmission. Common selection processes probe free virus transmission thus may not have detected antibodies targeting cell-cell transmission. Defining whether antibodies that preferentially target the cell-cell transmission exist, should aid resolution of the relative importance of this transmission mode and its inhibition.

Regardless of which scenario holds true, we would argue that cell-cell transmission and the ensuing virus production from infected cells cannot be scarce otherwise viremia levels would drop more dramatically during those periods when the autologous CD4bs specific neutralization response is effective and restricting free virus transmission. Of note, viral set points in chronic infection, while comparatively stable, nevertheless fluctuate, commonly within a 0.5 to 1 log range [108]. It is tempting to hypothesize that this fluctuation may be in part the result of alternating periods of effective neutralization of free virus by the autologous neutralization response, during which only cell-cell transmission occurs, followed by periods where the virus has escaped the neutralization response and both transmission modes are effective.

In sum our analyses provide compelling evidence that neutralizing antibodies, depending on their mode of action, differ in their capacity to block free virus and cell-cell transmission. According to current knowledge HIV relies on both transmission modes to maintain infection *in vivo*. We therefore argue that the efficacy of entry inhibitors and neutralizing antibodies to block cell-cell transmission needs to be considered.

## Materials and Methods

### Ethics statement

PBMC were purified from buffy coats from anonymous blood donations from healthy individuals obtained by the Zurich Blood Transfusion Service (http://www.zhbsd.ch/) under a protocol approved by the local ethics committee.

### Reagents

Properties and sources of antibodies and inhibitors used in this study are listed in Table S1. DARPin 57.2 was produced as described [109]. T-20 [110] was purchased from Roche Pharmaceuticals. Maraviroc [111] was purchased from Pfizer. CD4M47 was synthesized as described [45] and kindly provided by J. Robinson.

### Cells

293-T and HeLa cells were obtained from the American Type Culture Collection (ATCC). TZM-bl cells [34], A3.01 and A2.01 T-cells [112] were obtained from the NIH AIDS Research and Reference Reagent Program (NIH ARRRP). All adherent cell lines were cultivated in DMEM containing 10% heat inactivated FCS and antibiotics.

A3.01 cells endogenously express CD4 and CXCR4. The sister cell line A2.01 is CD4 negative. CCR5 expressing A3.01 cells (A3.01-CCR5) were generated using retroviral transduction as described ([41], C. Gordon, A. Trkola and J.P Moore unpublished data). Suspension cells were cultivated in RPMI containing 10% FCS and antibiotics.

Stimulated peripheral blood mononuclear cells (PBMC) from healthy blood donors were prepared as described [36] and cultivated in RPMI containing 10%FCS, 100 units per ml IL-2 and antibiotics.

### Virus preparation and concentration

Env encoding plasmids of subtype B Tier 1 isolates NL4-3 (X4) [113], SF162 (R5) [114] and 6535 (R5) [115] and Tier 2 JR-FL (R5) [116] were obtained from the NIH ARRRP.

Env–pseudotyped viruses were prepared by co-transfection of 293-T cells with plasmids encoding the respective Env gene and the luciferase reporter HIV vector pNLluc-AM [117] as described [36].

Env-pseudotyped particles (pp) generated with this vector are denoted Env^pp-lucAM^ (e.g. JR-FL^pp-lucAM^).

Where indicated the corresponding pNLgfp-AM pseudotyping vector (generated by P. Rusert and P. Ocampo) which encodes GFP instead of luciferase was used. Env- pseudotyped particles generated with this vector are denoted Env^pp-gfpAM^ (e.g. JR-FL^pp-gfpAM^).

Replication competent (rc) virus subtype B Tier-1 isolates ADA (R5), SF162 (R5), NL4-3 (X4), BZ167 (R5X4), Tier-2 isolates JR-FL (R5), JR-CSF (R5), ZA015, ZA016 and Tier-3 isolate ZA110 (R5) [53] were propagated on CD8-depleted PBMC and titered as described [118]. In experiments where replication competent and pseudotyped virus preparations are compared, viruses are denoted with rc and pp, respectively (e.g. JR-FL^rc^, JR-FL^pp^).

Replication competent virions were GFP labeled by two alternate procedures. We used the full length replication competent NL4-3 derived HIV-GagiGFP vector [13]. Alternatively, virions were labeled by incorporation of chimeric vpr-GFP as described [119]. To this end 293-T cells were co-transfected with a plasmid encoding a full length molecular clone of HIV (TN8 NL [120]) and the plasmid pEGFP-Vpr (gift from B. Paxton).

Alternatively, to obtain GFP labeled pseudoparticles, an Env gene deleted pseudotyping vector was generated from the full length replication competent HIV-GagiGFP vector [13]. Briefly, envelope from the HIV-GagiGFP construct was replaced by the corresponding env-deleted luciferase expressing sequence from pNLluc-AM via XhoI and EcoRI. This vector (HIV-iGFP) was then used to generate Env-pseudotyped particles by cotransfecting 293-T cells together with the desired envelope encoding plasmid. Env-pseudotyped particles generated with this vector are denoted Env^ppiGFP^ (e.g. JR-FL^ppiGFP^)

Replication competent β-lactamase labeled viral particles NL4-3^rc-BlaM^, were generated by co-transfecting the pCMV_4_-3BlaM-Vpr plasmid (gift from W. C. Greene), plasmid pAdVAntage (Promega) and the replication competent proviral vector TN8 as described [61]. To generate BlaM-vpr labeled JR-FL env pseudoviruses (JR-FL^pp-BlaM^) 293-T cells were co-transfected with plasmids pCMV_4_-3BlaM-Vpr, JR-FL env and pNLluc-AM.

All virus preparations were filtered upon harvesting and infectivity and/or p24 content determined to quantify input as described [118]. For the virus attachment and β-lactamase entry assays virus preparations were concentrated by ultracentrifugation (2 h at 4°C at 28'000 rpm; swing out rotor SW28, 32% sucrose cushion).

### Generation of TRIM5a expressing cells

Bicistronic lentiviral GFP and TRIM5α expression vectors huTRIM5α or rhTRIM5α [51] were provided by J.L.Riley. Lentiviral vectors were produced upon co-transfection of 293-T cells with the TRIM5α encoding vector, the VSV envelope encoding plasmid pHEF-VSVG [121] obtained through the NIH ARRRP) and the packaging plasmid pCMV-dR8.91 ([122]; gift from D. Trono). A3.01-CCR5 cells were transduced by spinoculating (2 h at 1200 g) 100 lentiviral particles per cells in DMEM containing 10% FCS, antibiotics, and 10 µg/ml DEAE. PBMC were transduced one day after isolation and stimulation by spinoculation (2 h 1200 g) with 800 lentiviral particles in RPMI containing 10% FCS, antibiotics and 8 ug/ml Polybrene. PBMC Transduced PBMC were cultured on 48 wells coated with OKT3 and 2 µg/ml CD28 and TRIM5α positive cells were retrieved by FACS sorting on day 4 after transduction. Expression of huTRIM5α or rhTRIM5α was monitored by detection of bicistronic expressed GFP by FACS.

### Assessment of free virus and cell-cell transmission in the PBMC^HIV+^/TZM-bl infection system

We developed an assay system based on infection of TZM-bl cells, which allows easy and quantitative discrimination between cell-free and cell-cell transmission. This is possible as many R5 viruses depend on polycationic supplements in the cell culture medium in order to infect TZM-bl cells as cell-free virions but not during cell-cell transmission (Fig. 1).

For cell-free virus infection, the neutralization activity of mAbs and inhibitors was evaluated on TZM-bl cells essentially as described using replication competent virus as inoculum [36]. Cell-free, replication competent virus input was chosen to yield virus infectivity corresponding to 5'000–10'000 relative light units (RLU) per 96 well in absence of inhibitors. Cell-free virus infections were carried out in culture medium containing 10 µg/ml of the polycation DEAE (diethylaminoethyl; Amersham Biosciences, Fairfield, Connecticut, USA) if not otherwise indicated.

To assess cell-cell transmission and inhibition thereof, stimulated CD8-depleted peripheral blood mononuclear cells (PBMC) from healthy blood donors were infected with replication competent virus stocks at a MOI 0.01. Cell-cell transmission in the PBMC^HIV+^/TZM-bl infection system had the same linear dynamic range as cell-free transmission (Fig. 1A and B). At the highest virus or infected cell input a reduction in luciferase reporter signal is observed due to increased cell death and the resultant loss of infected TZM-bl cells. Input of infected cells was chosen as such that ensuing infection of TZM-bl cells was in the same range as in the free virus infections (virus infectivity corresponding to 5'000–10'000 relative light units (RLU) per well in absence of inhibitors). To ensure that cell-cell transmission is always probed in the linear range of the assay, a titration of donor cell input, as depicted in Fig. 1A, was included in each individual experiment. Cell-cell virus infections were performed in culture medium containing no DEAE if not otherwise indicated. On day 4 post infection, infected PBMC were washed twice to remove free virions. Cells were then pre-incubated with virus directed inhibitors for 1 h before co-culturing with TZM-bl target cells (1×10^4^ per well). To assess activity of target cell directed agents, TZM-bl cells were pre-incubated with inhibitors before co-culturing with infected PBMC. 48 hours after infection cells were lysed and luciferase reporter gene production measured upon addition of firefly luciferase substrate (Promega, Madison Wisconsin, USA). Inhibitor and antibody concentrations causing 50% reduction in viral infectivity (50% inhibitory concentration, IC50) were calculated by fitting pooled data from three to four independent experiments to sigmoid dose response curves (variable slope) using GraphPad Prism. If 50% inhibition was not achieved at the highest drug concentration a greater-than value was recorded.

Only R5 viruses which we determined to depend upon DEAE-Dextran to efficiently infect TZM-bl cells as free virus inoculum were used in the PBMC^HIV+^/TZM-bl assay (Fig. S2). For these isolates no infection was detectable over a wide range of virus input in the absence of DEAE-Dextran. Although this dependence can be overcome at very high virus concentrations, infectivity remained 1–2 orders of magnitude lower. Levels of virus and infected cell input that mediate efficient cell-cell transmission but restrict free virus infectivity were then employed in the assays. R5 and X4 using virus strains which efficiently infect TZM-bl cells in the absence of cationic compounds cannot be used in this assay. While DEAE-Dextran also improves infectivity of these viruses (Fig. S2), residual infectivity in the absence of the polycation is too high and impedes precise discrimination of cell-free and cell-cell infection in the DEAE dependent PBMC^HIV+^/TZM-bl infection system.

### Assessment of free virus and cell-cell transmission using envelope pseudotyped virus particles

We utilized the DEAE dependent TZM-bl infection assay system also to assess single-round virus infection during cell-cell transmission using envelope pseudotyped viruses. The pseudotype backbone used in these experiments (pNLgfp-AM) does not encode for luciferase, which allowed discrimination between donor and target cell infection, as only in the TZM-bl target cells luciferase production will be induced upon infection. Free virus infection in presence of DEAE was performed as described with minor modifications [36]. Cell-free virus input was chosen to yield virus infectivity corresponding to 5'000–10'000 relative light units (RLU) per 96 well in absence of inhibitors. Virus and inhibitors were preincubated for 1 h at 37°C in 96 well plates, then TZM-bl (10^4^ per well) were added. To assess the neutralization activity of mAbs and inhibitors during cell-cell transmission of pseudovirus, 293-T cells (10^4^ per well) were seeded in 96-well plates, and 24 h later transfected with 24 µg of the pseudovirus backbone pNLgfp-AM and 8 µg of the JR-FL env per plate (0.33 µg per well) - using polyetheylenimine (PEI, linear 25 kDa, Polysciences) as transfection agent. Twenty-four hours post transfection virus producing 293-T cells were washed twice with DMEM (10% FCS, P/S) and pre-incubated with virus directed inhibitors (1 h at 37°C). Then TZM-bl cells (10^4^ per well) were added. No DEAE Dextran was present in the cell-cell transmission setting. After 48 hours of co-culture, infection of the TZM-bl cells was monitored by quantifying the production of the reporter luciferase and the 50% inhibitory concentration (IC50) of the respective drugs was assessed.

### Assessment of free virus and cell-cell transmission using rhTRIM5α restricted A3.01-CCR5 cells

The neutralization activity of mAbs and inhibitors was additionally evaluated on cells expressing rhesus (rh) TRIM5α as they have been shown to restrict preferentially free virus transmission (Figure S3B and [51]). A3.01-CCR5 were transduced with human or rhTRIM5α or mock treated and used as target cells in T-T cell transmission experiments and were co-cultivated with infected A3.01-CCR5^HIV+^. To study free virus infection the same set of target cells were infected with cell-free replication competent virus. TRIM5α expression was monitored by the expression of bi-cistronic expressed GFP. HIV infection of cells was detected by intracellular p24 staining by FACS using the BD Cytofix/Cytoperm Fixation and Permeabilization Kit (BD Biosciences) and mAb KC57-RD1 (anti-HIV-1 p24-Gag, Beckman Coulter), following the manufacturers' instructions. To assess the influence of rhTRIM5α on cell-cell transmission, A3.01-CCR5^huTRIM5α^, A3.01-CCR5^rhTRIM5α^ or mock treated A3.01-CCR5 cells and either co-cultured with infected donor cells (A3.01-CCR5^HIV+^) or cell-free virus 6 days post transduction. The same virus stocks were used for free virus infections and to infect donor cells. Infection was monitored after 2–7 days of culture by measuring Gag protein expression in the target cell population. To monitor influence of the transmission mode on entry inhibition cell-cell transmission was assessed by determining efficacy of inhibition using A3.01-CCR5^rhTRIM5α^ and infected donor cells (A3.01-CCR5^HIV+^). This was compared to inhibition of free virus infection of mock treated A3.01-CCR5 cells. Virus input for both transmission modes was adjusted to yield a comparable output of approximately 10% Gag positive A3.01-CCR5 cells in absence of inhibitors. Inhibitor concentrations which yield maximum inhibition of cell-free virus infection were determined for all compound/virus pairings and probed at these doses in the cell-cell transmission setting. Virus directed inhibitors were preincubated with cell-free replication competent virus or infected A3.01-CCR5 cells (50'000 per 96 well) for 1 h at 37°C. A3.01-CCR5^rhTRIM5α^ target cells were added (50'000 per well) and infection allowed to spread for 2–7 days depending on the growth kinetics of the respective isolates. Infectivity was assessed by intracellular p24 staining. % inhibition = 100−100/[% infected cells in uninhibited sample] * [% infected cells in sample x]. As control, infection with cell-free and cell-associated virus was performed using transwell chambers (12-well 0.4 µm polyester-membrane dishes (Corning Life Sciences, Corning, NY) and virus inocula (cell-free or cell- associated) added to the transwell insert. Uninfected human or rhesus TRIM5α transduced A3.01-CCR5 were seeded as target cells in the bottom chamber.

### Assessment of free virus and cell-cell transmission using rhTRIM5α restricted PBMC

The PBMC^HIV+^/PBMC^rhTRIM5α^ transmission assays were performed essentially as described for A3.01-CCR5 cells. To assess cell-cell transmission using rhTRIM5α, stimulated, CD8 depleted PBMC were transduced with rhTRIM5α one day after isolation, sorted 4 days post-transduction and co-cultivated with infected PBMC one day after sorting. In parallel cells were mock treated and cell-free inhibition was monitored utilizing the same virus stocks. Infection was monitored after 3 days of culture by measuring Gag protein expression in the target cell population.

### Virus attachment assay

Vpr-GFP or Gag-iGFP labeled virion attachment to target cells was studied in presence or absence of entry inhibitors. GFP labeled viruses were preincubated with virus-directed antibodies or inhibitors for 1 h at 37°C, then added to wells of a 96-well round-bottom plates containing target cells (PBMC (100'000 cells/well); A3.01-CCR5, A2.01, HeLa, and TZM-bl: (50'000 cells/well)) in a total volume of 100 µl. Attachment of virus to target cells was synchronized by spinoculation (2 h at 1200 g) at 23°C [43]. This low temperature allows efficient attachment of virions to target cells and receptor engagement but impedes virus-cell fusion [123]. Following spinoculation, unbound virus was removed by washing cells twice in FACS buffer (PBS, 2% FBS, 0.1% azide). Cells were then fixed in 1.5% paraformaldehyde (PFA) and GFP positive cells indicative of virus attachment quantified by flow cytometry on a CyAn ADP instrument (Beckman Coulter). Data analysis was performed with FlowJo software (Treestar). The endogenous green fluorescence of mock treated cells (no virus) was determined and the mean fluorescence intensity (MFI) of virus treated samples corrected for this value. 100% attachment (0% inhibition, medium control) was determined in cells treated with virus in absence of inhibitors. The inhibition achieved by the various inhibitors and neutralizing antibodies was expressed relative to this value. % inhibition of attachment = 100−100/[MFI medium control] * [MFI inhibitor x].

### Virus fusion assay

We employed a virion-based fusion assay, which detects the enzymatic activity of virion co-packaged β-lactamase post fusion, to assess virus entry essentially as described previously [60], [61]. Virus entry is thereby measured as the extent of cleavage of a cytosolic, fluorogenic substrate by virion co-packaged β-lactamase (BlaM). The latter is achieved by incorporation of chimeric BlaM-vpr into viral particles which is delivered to the target cells cytosol upon successful entry [61]. To test the pre-attachment inhibitory potency of mAbs and inhibitors, BlaM-Vpr containing viruses were preincubated with nAbs or inhibitors for 1 h at 37°C. Target cells (PBMC/100'000 cells/well or A3.01-CCR5/50'000 cells/well) were added and virus attachment initiated by spinoculation (2 h, 1'200 g, 23°C). Cells were then immediately washed with CO_2_-independent medium (Gibco) to remove unbound virus and inhibitors. In parallel, to test post attachment activity of entry inhibitors, the inhibitors or nAbs were added after spinoculation (after excess unbound virus had been washed off) and were incubated with the virion bearing cells for 1 h at 23°C. To initiate virus–cell fusion, samples from both the pre and post attachment conditions, were incubated for 3 h at 37°C. The cells were then washed once in medium and loaded with the fluorogenic β-lactamase substrate CCF2/AM (Invitrogen) and incubated for 1 h at room temperature following the manufacturer's instructions. Cells were then washed twice in developing medium (CO_2_-independent medium (Gibco), 2.5 mM probenecid (Sigma), 10% FBS) and incubated overnight at room temperature to allow the β-lactamase to cleave CCF2/AM. Following a wash step with PBS, cells were stained with anti-CD4-APC (Caltag), washed again and fixed in 3% paraformaldehyde. CD4 positive cells were accounted for by flow cytometry and cell populations containing uncleaved CCF2/AM (520 nm, no virus fusion) and cleaved CCF2/AM (447 nm; virus fusion) determined. Inhibition of fusion was determined by reduction in cell numbers positive for cleaved CCF2/AM (447 nm, 450/50 filter) fluorescence and calculated as % inhibition = 100−100/[% infected cells in medium control] * [% infected cells in inhibitor×treated sample].

### Virus entry assay based of luciferase reporter gene assay

To analyze post-attachment activity of mAbs and inhibitors, we studied infection of A3.01-CCR5 cells by Env-pseudotyped luciferase reporter viruses. To test pre-attachment activity, inhibitors and mAbs were pre-incubated for 1 h at 37°C, and then spinoculated onto A3.01-CCR5 cells as described above. Unbound virus and inhibitors were washed off immediately after spinoculation. To test post-attachment activity, virus was first spinoculated onto A3.01-CCR5 cells, residual virus washed off and then inhibitors incubated with the virus bearing cells for 1 h at 23°C. Both pre- and post-attachment cultures were then cultivated for 48 h at 37°C before infection was monitored by determining luciferase production as described above.

## Supporting Information

Figure S1 **Dependence of R5 viruses on DEAE-Dextran during cell-free transmission.** (**A**) Free virus released from infected donor cells during cell-cell transmission has no impact on assessment of cell-cell transmission. JR-FL infected PBMC were co-cultured with HeLa cells (CD4 and CCR5 negative) to mimic co-culture condition in the PBMC^HIV+^/TZM-bl infection system without allowing cell-cell transmission to occur. Supernatant was harvested at the indicated time points, transferred onto TZM-bl cells and assessed for infectivity in absence of DEAE-Dextran. During the 48 h co-culture period only minute amounts of virus are released from the infected PBMC which fail to infect in the absence of DEAE-Dextran. Thus, at the chosen infected cell input, virus transmission in the PBMC^HIV+^/TZM-bl infection system in absence of DEAE-Dextran occurred almost exclusively through cell-cell transmission. Data are derived from one of two independent experiments. Means and SEM of triplicate samples are shown. (**B**) Cell-cell transmission is more rapid than cell-free transmission. Cell-cell transmission of JR-FL from infected PBMC to TZM-bl in absence of DEAE Dextran (left panel) and cell-free JR-FL infection of TZM-bl in presence of 10 µg/ml DEAE-Dextran (right panel) was monitored at the indicated time points by determining luciferase reporter production (RLU). Data points are means of triplicate measurements. Bars represent SEM.
(TIF)

Figure S2 **R5 viruses differ in their DEAE-Dextran dependence during cell-free transmission.** **(A) DEAE-Dextran dependent cell-free infection of TZM-bl cells by R5 viruses** TZM-bl cells were infected with serial dilutions of cell-free R5 virus isolates (ADA, ZA110, ZA015 and ZA016) in presence (black squares) or absence (red squares) of 10 µg/ml DEAE-Dextran. Infection was determined by measuring luciferase production after 48 h (recorded as RLU). Each virus dilution was probed in quadruplicates. Bars represent SEM. One of two independent experiments is shown. **(B) Absence of DEAE-Dextran as media supplement has no effect on cell-cell transmission of HIV-1 to TZM-bl cells.** Serial dilutions of PBMC infected with different R5 isolates (ADA, ZA110, ZA015 and ZA016) were incubated with TZM-bl cells in presence (black circles) or absence (red circles) of DEAE-Dextran. Infection was determined by measuring luciferase production after 48 h (recorded as RLU). Each infected cell input was probed in triplicate. Error bars represent SEM. One of two independent experiments is shown. **(C) DEAE-Dextran independent cell-free infection of TZM-bl cells by certain R5 and X4 using viruses.** TZM-bl cells were infected with serial dilutions of cell-free R5 virus isolates JR-CSF and SF162, the R5X4 virus BZ167 and the X4 strain NL4-3 in presence (black squares) or absence (red squares) of DEAE-Dextran. Infection was determined by measuring luciferase production after 48 h (recorded as RLU). Each virus dilution was probed in quadruplicates. Bars represent SEM. One of two independent experiments is shown.
(TIF)

Figure S3 **Rhesus TRIM5α restriction allows precise dissection of cell-free and cell-cell transmission of HIV-1.** **(A) Rhesus TRIM5α transduced cells are highly resistant to cell-free single round and multiple round infection.** Infection of rhesusTRIM5α or mock transduced A3.01-CCR5 cells with the indicated env-pseudotyped, luciferase reporter viruses (left panel) or replication competent SF162 isolate (right panel). Infection of the reporter virus was determined by measuring luciferase production after 48 h (recorded as RLU/ml). Infection of SF162 was monitored by determining p24 antigen production. Both cell-free infection with single round, env pseudotyped virus and replication competent virus isolates proved to be almost completely restricted in rhTRIM5α transduced A3.01-CCR5 cells. One of two independent experiments for each virus isolate is shown. Error bars represent SEM. **(B) Cell-cell transmission overcomes rhTRIM5α mediated restriction of HIV-1.** Uninfected or SF162-infected A3.01-CCR5 cells (donors) were co-cultivated with the indicated A3.01-CCR5 target cells (mock treated (no gfp), rhTRIM5α (gfp positive), huTRIM5α (gfp positive)) either in direct coculture (left panel or separated by transwells (right panel). Infection was assessed by intracellular HIV-1 Gag staining after 6 days of coculture. Data show one representative out of three independent experiments. **(C) Cell-cell transmission but not enforced contact between virus and target cell overcomes rhTRIM5α mediated entry restriction.** Comparison of the infectivity of cell-free SF162 infection of i) spinoculated, ii) magnetic bead bound virus and iii) virus added without enforced adsorption with cell-cell transmission (direct cocultivation and transwell). Infection of mock treated, rhTRIM5α and huTRIM5α A3.01-CCR5 target cells was investigated. One representative out of three independent experiments is depicted. To allow comparison, data are normalized to infection levels obtained by spinoculating cell-free SF162 onto mock transduced cells.
(TIF)

Figure S4 **Efficient inhibition of Cell-Cell transmission by V3 directed antibodies.** **(A) V3 directed antibody 1–79 efficiently inhibits cell-cell transmission of replication competent SF162.** Activity of V3 loop mAb 1–79 and CD4bs directed mAb b12 to inhibit cell-cell transmission was studied by co-cultivating rhTRIM5α transduced TZM-bl with SF162^rc^ infected PBMC (red circles; no DEAE in infection media). Inhibition of free virus transmission of SF162^rc^ was monitored in parallel on TZM-bl target cells in absence of rhTRIM5α (black squares; 10 µg/ml DEAE in infection media). Infection was determined by measuring luciferase production after 48 h (recorded as RLU). Lines depict fitted results derived from three independent experiments in which each sample condition was performed in duplicates. Error bars depict SEM. **(B) Single round infection by 6535 is sensitive to 447-52D inhibition during cell-cell transmission.** Activity of V3 loop mAb 447-52D and CD4bs directed b12 to inhibit cell-cell transmission was studied by co-cultivating rhTRIM5α transduced TZM-bl with 6535 pseudovirus transfected 293-T cells (red circles; no DEAE in infection media). Inhibition of free virus transmission of cell-free 6535^pp-lucAM^ was monitored in parallel on TZM-bl target cells in absence of rhTRIM5α (black squares; 10 µg/ml DEAE in infection media). Infection was determined by measuring luciferase production after 48 h (recorded as RLU). Lines depict fitted results derived from three independent experiments in which each sample condition was performed in duplicates. Error bars depict SEM.
(TIF)

Table S1 **Origin and specificity of mAbs and inhibitors.** This table lists the origin and specificity of all monoclonal antibodies and inhibitors used in the current study.
(PDF)

Table S2 **Antibody and inhibitor concentrations.** This table lists the individual antibody and inhibitor concentrations used in experiments depicted in Figures 4 to [Fig ppat-1002634-g005] [Fig ppat-1002634-g006] 7.
(PDF)
