# Supplementary figures and images for: Cell-Cell Transmission Enables HIV-1 to Evade Inhibition by Potent CD4bs Directed Antibodies
Source: PLoS Pathog. 2012 Apr 5;8(4):e1002634. doi: 10.1371/journal.ppat.1002634 (PMC3320602; doi:10.1371/journal.ppat.1002634)

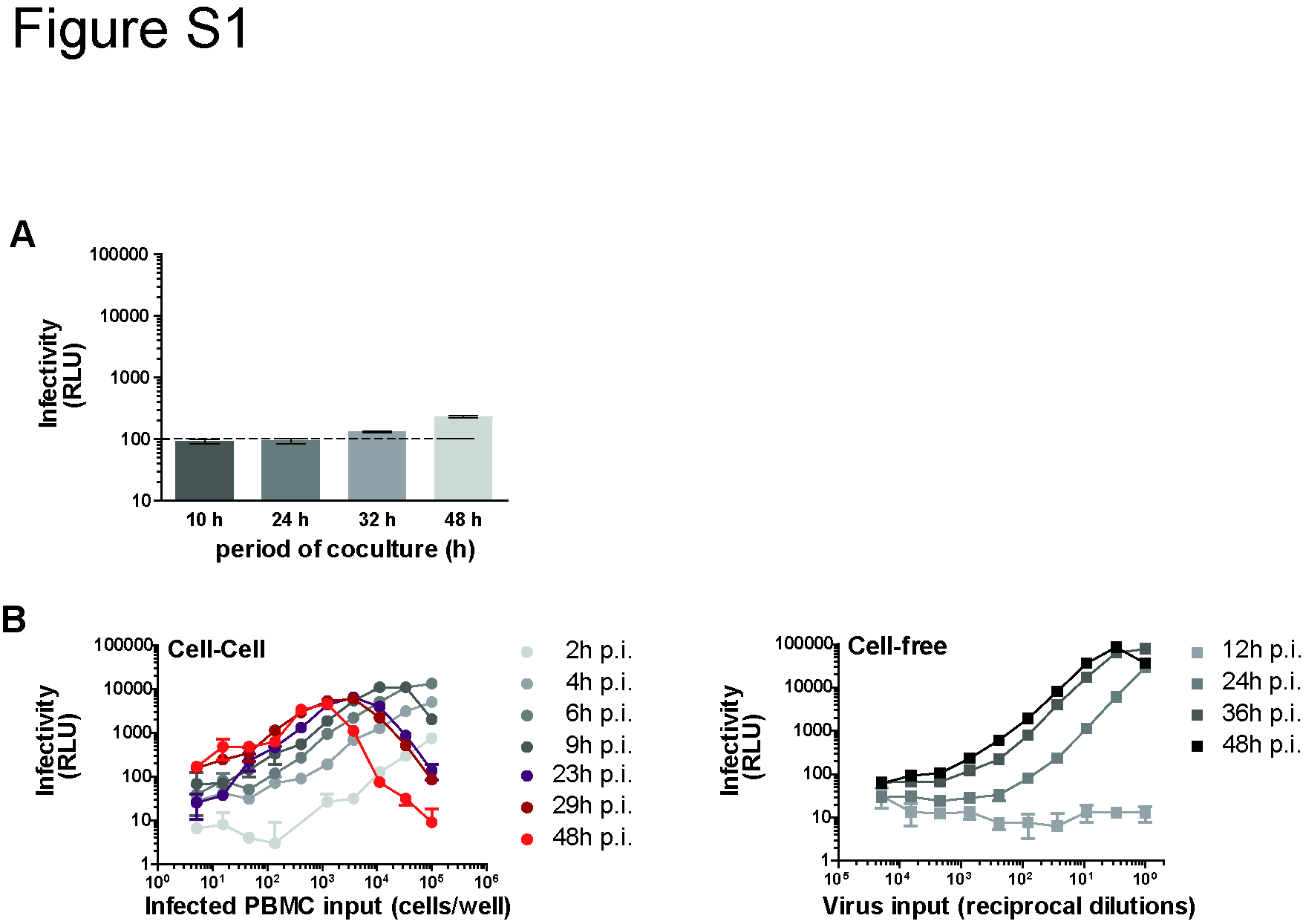

Supplement: Figure S1 — Dependence of R5 viruses on DEAE-Dextran during cell-free transmission. (A) Free virus released from infected donor cells during cell-cell transmission has no impact on assessment of cell-cell transmission. JR-FL infected PBMC were co-cultured with HeLa cells (CD4 and CCR5 negative) to mimic co-culture condition in the PBMCHIV+/TZM-bl infection system without allowing cell-cell transmission to occur. Supernatant was harvested at the indicated time points, transferred onto TZM-bl cells and assessed for infectivity in absence of DEAE-Dextran. During the 48 h co-culture period only minute amounts of virus are released from the infected PBMC which fail to infect in the absence of DEAE-Dextran. Thus, at the chosen infected cell input, virus transmission in the PBMCHIV+/TZM-bl infection system in absence of DEAE-Dextran occurred almost exclusively through cell-cell transmission. Data are derived from one of two independent experiments. Means and SEM of triplicate samples are shown. (B) Cell-cell transmission is more rapid than cell-free transmission. Cell-cell transmission of JR-FL from infected PBMC to TZM-bl in absence of DEAE Dextran (left panel) and cell-free JR-FL infection of TZM-bl in presence of 10 µg/ml DEAE-Dextran (right panel) was monitored at the indicated time points by determining luciferase reporter production (RLU). Data points are means of triplicate measurements. Bars represent SEM. (TIF) [file ppat.1002634.s001.tif]

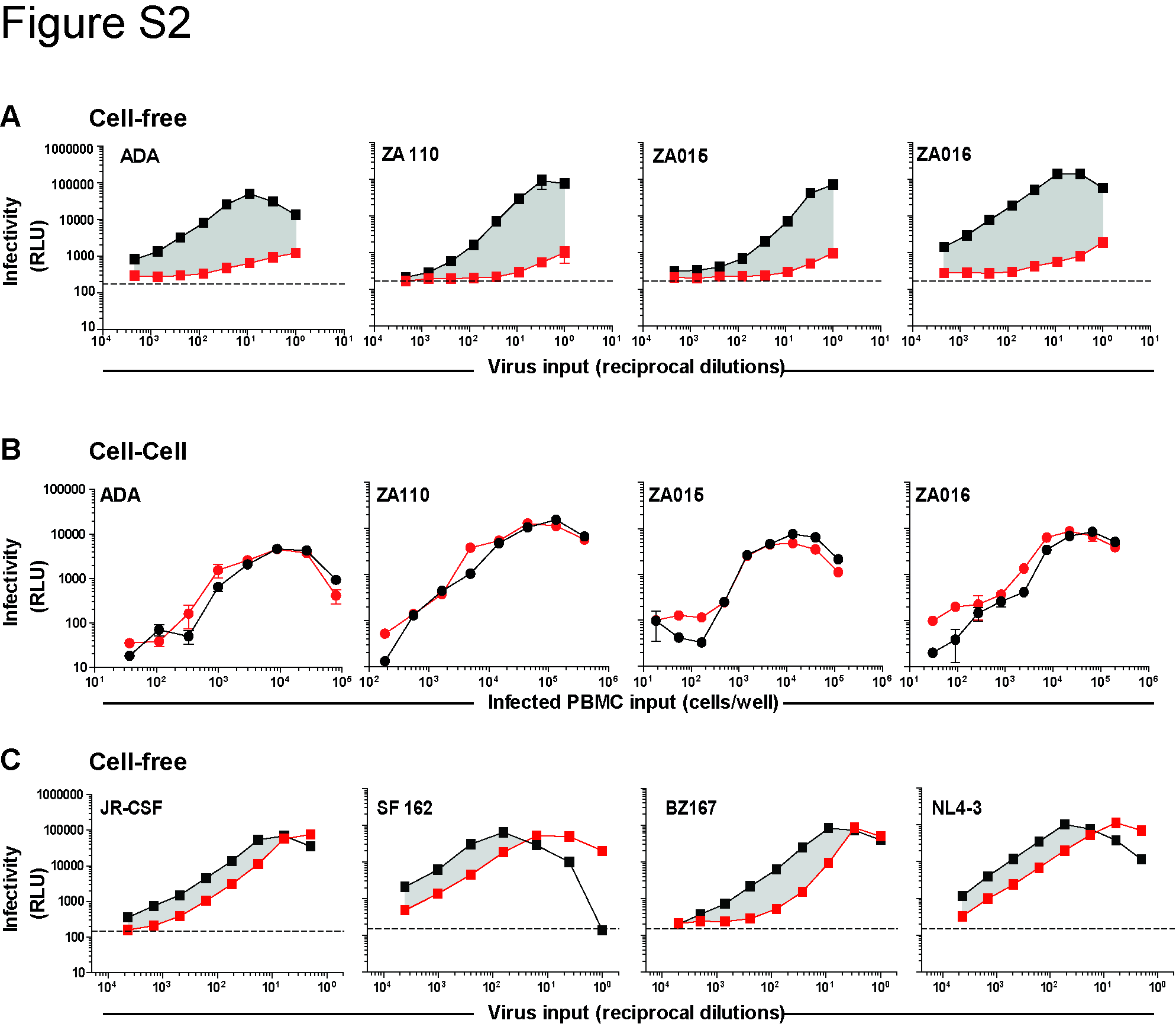

Supplement: Figure S2 — R5 viruses differ in their DEAE-Dextran dependence during cell-free transmission. (A) DEAE-Dextran dependent cell-free infection of TZM-bl cells by R5 viruses TZM-bl cells were infected with serial dilutions of cell-free R5 virus isolates (ADA, ZA110, ZA015 and ZA016) in presence (black squares) or absence (red squares) of 10 µg/ml DEAE-Dextran. Infection was determined by measuring luciferase production after 48 h (recorded as RLU). Each virus dilution was probed in quadruplicates. Bars represent SEM. One of two independent experiments is shown. (B) Absence of DEAE-Dextran as media supplement has no effect on cell-cell transmission of HIV-1 to TZM-bl cells. Serial dilutions of PBMC infected with different R5 isolates (ADA, ZA110, ZA015 and ZA016) were incubated with TZM-bl cells in presence (black circles) or absence (red circles) of DEAE-Dextran. Infection was determined by measuring luciferase production after 48 h (recorded as RLU). Each infected cell input was probed in triplicate. Error bars represent SEM. One of two independent experiments is shown. (C) DEAE-Dextran independent cell-free infection of TZM-bl cells by certain R5 and X4 using viruses. TZM-bl cells were infected with serial dilutions of cell-free R5 virus isolates JR-CSF and SF162, the R5X4 virus BZ167 and the X4 strain NL4-3 in presence (black squares) or absence (red squares) of DEAE-Dextran. Infection was determined by measuring luciferase production after 48 h (recorded as RLU). Each virus dilution was probed in quadruplicates. Bars represent SEM. One of two independent experiments is shown. (TIF) [file ppat.1002634.s002.tif]

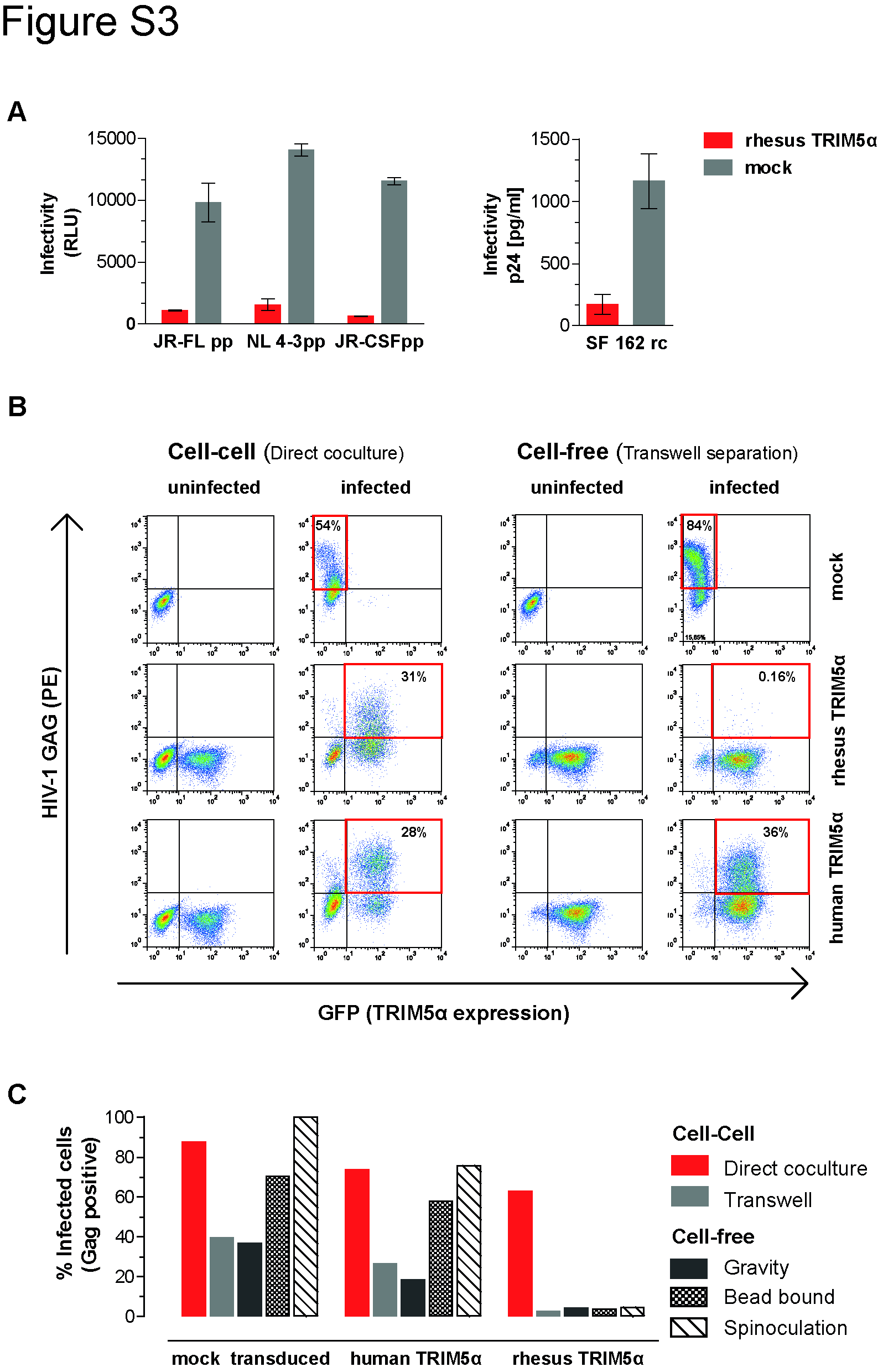

Supplement: Figure S3 — Rhesus TRIM5α restriction allows precise dissection of cell-free and cell-cell transmission of HIV-1. (A) Rhesus TRIM5α transduced cells are highly resistant to cell-free single round and multiple round infection. Infection of rhesusTRIM5α or mock transduced A3.01-CCR5 cells with the indicated env-pseudotyped, luciferase reporter viruses (left panel) or replication competent SF162 isolate (right panel). Infection of the reporter virus was determined by measuring luciferase production after 48 h (recorded as RLU/ml). Infection of SF162 was monitored by determining p24 antigen production. Both cell-free infection with single round, env pseudotyped virus and replication competent virus isolates proved to be almost completely restricted in rhTRIM5α transduced A3.01-CCR5 cells. One of two independent experiments for each virus isolate is shown. Error bars represent SEM. (B) Cell-cell transmission overcomes rhTRIM5α mediated restriction of HIV-1. Uninfected or SF162-infected A3.01-CCR5 cells (donors) were co-cultivated with the indicated A3.01-CCR5 target cells (mock treated (no gfp), rhTRIM5α (gfp positive), huTRIM5α (gfp positive)) either in direct coculture (left panel or separated by transwells (right panel). Infection was assessed by intracellular HIV-1 Gag staining after 6 days of coculture. Data show one representative out of three independent experiments. (C) Cell-cell transmission but not enforced contact between virus and target cell overcomes rhTRIM5α mediated entry restriction. Comparison of the infectivity of cell-free SF162 infection of i) spinoculated, ii) magnetic bead bound virus and iii) virus added without enforced adsorption with cell-cell transmission (direct cocultivation and transwell). Infection of mock treated, rhTRIM5α and huTRIM5α A3.01-CCR5 target cells was investigated. One representative out of three independent experiments is depicted. To allow comparison, data are normalized to infection levels obtained by spinoculating cell-free SF162 onto [file ppat.1002634.s003.tif]

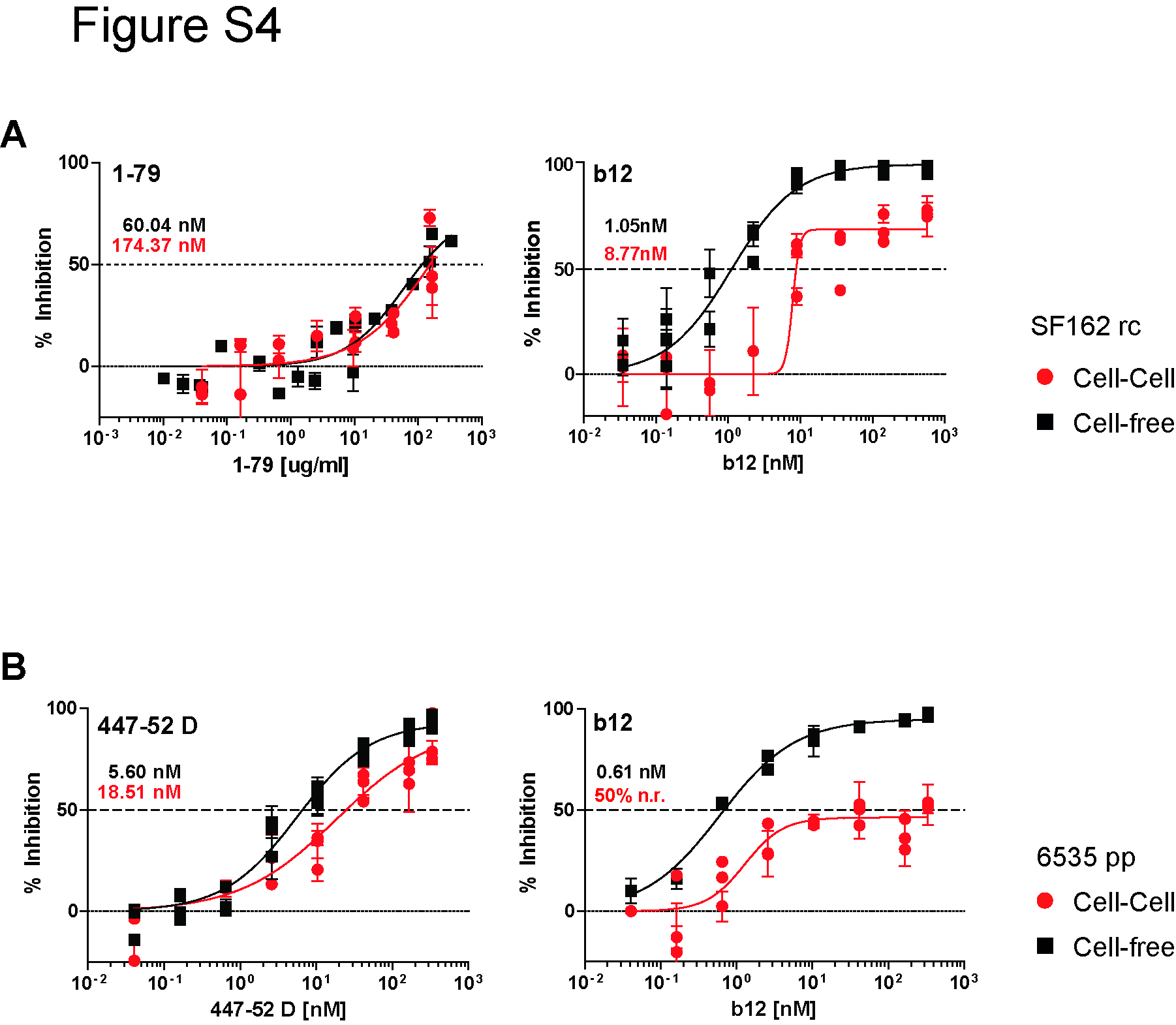

Supplement: Figure S4 — Efficient inhibition of Cell-Cell transmission by V3 directed antibodies. (A) V3 directed antibody 1–79 efficiently inhibits cell-cell transmission of replication competent SF162. Activity of V3 loop mAb 1–79 and CD4bs directed mAb b12 to inhibit cell-cell transmission was studied by co-cultivating rhTRIM5α transduced TZM-bl with SF162rc infected PBMC (red circles; no DEAE in infection media). Inhibition of free virus transmission of SF162rc was monitored in parallel on TZM-bl target cells in absence of rhTRIM5α (black squares; 10 µg/ml DEAE in infection media). Infection was determined by measuring luciferase production after 48 h (recorded as RLU). Lines depict fitted results derived from three independent experiments in which each sample condition was performed in duplicates. Error bars depict SEM. (B) Single round infection by 6535 is sensitive to 447-52D inhibition during cell-cell transmission. Activity of V3 loop mAb 447-52D and CD4bs directed b12 to inhibit cell-cell transmission was studied by co-cultivating rhTRIM5α transduced TZM-bl with 6535 pseudovirus transfected 293-T cells (red circles; no DEAE in infection media). Inhibition of free virus transmission of cell-free 6535pp-lucAM was monitored in parallel on TZM-bl target cells in absence of rhTRIM5α (black squares; 10 µg/ml DEAE in infection media). Infection was determined by measuring luciferase production after 48 h (recorded as RLU). Lines depict fitted results derived from three independent experiments in which each sample condition was performed in duplicates. Error bars depict SEM. (TIF) [file ppat.1002634.s004.tif]
