# Supplementary material for: Cell-Cell Transmission Enables HIV-1 to Evade Inhibition by Potent CD4bs Directed Antibodies
Source: PLoS Pathog. 2012 Apr 5;8(4):e1002634. doi: 10.1371/journal.ppat.1002634 (PMC3320602; doi:10.1371/journal.ppat.1002634)
Supplement: Table S1 — Origin and specificity of mAbs and inhibitors. This table lists the origin and specificity of all monoclonal antibodies and inhibitors used in the current study. (PDF) [file ppat.1002634.s005.pdf]

**Supplementary Table 1: Origin and specificity of mAbs and inhibitors**

| Inhibitor | Drug class | Target | Epitope (HxB2) / Binding site | MW | Reference | Source |
|---|---|---|---|---|---|---|
| **CD4-IgG2** | tetrameric CD4 molecule | gp120 | CD4bs | 175 kDa | Allaway et al.1995 *AIDS Res Hum Retroviruses.11(5):533-9.* | W.Olson, Progenics |
| **CD4M47** | miniprotein mimetic | gp120 | CD4bs | 3 kDa | Stricher et al. 2008 *J Mol Biol, 2008. 382(2): 510-24* | J.Robinson |
| **VRC01** | Antibody | gp120 | CD4bs | 150 kDa | Wu, Yang et al. 2010 *Science.329(5993):856-61* | J.Mascola |
| **b12** | Antibody | gp120 | CD4bs | 150 kDa | Barbas et al. 1992 *PNAS.89(19):9339-43* | D.Burton |
| **1F7** | Antibody | gp120 | CD4bs | 150 kDa | Buchacher et al.1994 *AIDS Res Hum Retroviruses.10(4):359-69*<br>Kunert et al.1998 *AIDS Res Hum Retroviruses. 14(13):1115-28* | H.Katinger, Polymun |
| **2G12** | Antibody | gp120 | carbohydrate | 150 kDa | Trkola et al.1996 *J Virol.70(2):1100-8* | H.Katinger, Polymun |
| **447-52D** | Antibody | gp120 | V3 (312-315) | 150 kDa | Gorny et al.1992 *J Virol.66(12):7538-42* | S. Zolla-Pazner, NIH ARRRP |
| **1-79** | Antibody | gp121 | V3 (312-315) | 151 kDa | Scheid et al. 2009. *Nature*. 458:636–640. | M. Nussenzweig |
| **2F5** | Antibody | gp41 | MPER (662-667) | 150 kDa | Muster et al.1993 *J Virol.67(11):6642-7* | H.Katinger, Polymun |
| **4E10** | Antibody | gp41 | MPER (671-676) | 150 kDa | Stiegler et al. 2001 *AIDS Res Hum Retroviruses. 10;17(18):1757-65*<br>Zwick et al. 2001 *J Virol.75(22):10892-905* | H.Katinger, Polymun |
| **T-20 (Fuzeon)** | Peptide, fusion inhibitor | gp41 | Fusion peptide | 4.5 kDa | Wild et al.1993 *AIDS Res Hum Retroviruses. 9(11):1051-3* | Purchased from Roche Pharmaceuticals |
| **DARPin 57.2** | Designed Ankyrin Repeat Protein | CD4 | CD4 domain 1 | 18 kDa | Schweizer, Rusert et al. 2008 *PLoS Pathog. 4(7):e1000109* | A.Trkola |
| **OKT4a** | Antibody | CD4 | CD4 domain 1 | 150 kDa | Nicholson et al.1986 *The Journal of Immunology. 137(1): 323-329.* | Orthomune |
| **13B8.2** | Antibody | CD4 | CD4 domain 1 | 150 kDa | Benkirane et al.1993. *EMBO J. 242(1): 233-237.* | Q. Sattentau |
| **AD101 (SCH-350581)** | Small molecule inhibitor | CCR5 | Tansmembrane region of CCR5 | 0.5 kDa | Strizki, Xu et al. 2001 *PNAS. 98(22):12718-23* | J.Stritzki, Schering Plough |
| **Maraviroc** | Small molecule inhibitor | CCR5 | Transmembrane region of CCR5 | 0.5 kDa | Dorr, Westby et al. 2005 *Antimicrob Agents Chemother. 49(11):4721-32* | Purchased from Pfizer |
| **PRO 140** | Antibody | CCR5 | Nt and ECL2 domains | 150 kDa | Olson, Rabut et al. 1999 *J Virol.73(5):4145-55* | W.Olson, Progenics |
| **PSC-RANTES** | Chemokine derivative | CCR5 | ECL2 | 8 kDa | Hartley, Gaertner et al. 2004 *PNAS.101(47)16460-16465* | O.Hartley |
