# Supplementary material for: Cell-Cell Transmission Enables HIV-1 to Evade Inhibition by Potent CD4bs Directed Antibodies
Source: PLoS Pathog. 2012 Apr 5;8(4):e1002634. doi: 10.1371/journal.ppat.1002634 (PMC3320602; doi:10.1371/journal.ppat.1002634)
Supplement: Table S2 — Antibody and inhibitor concentrations. This table lists the individual antibody and inhibitor concentrations used in experiments depicted in Figures 4 to 7. (PDF) [file ppat.1002634.s006.pdf]

Table S2. Antibody and inhibitor concentrations

	Figure 4	Figure 4 and 6	Figure 7
CD4-IgG2	50 ug/ml	50 ug/ml	5 ug/ml
CD4M47	-	-	5 ug/ml
b12	50 ug/ml	50 ug/ml	10 ug/ml
VRC01	-	50 ug/ml	-
2F5	100 ug/ml	100 ug/ml	100 ug/ml
4E10	100 ug/ml	100 ug/ml	100 ug/ml
T-20	5 ug/ml	10	5 ug/ml
DARPin 57.2	-	-	1 uM
OKT4a	-	-	10 ug/ml
13B8.2	-	-	50 ug/ml
PRO 140	-	-	100 ug/ml
AD101	-	-	10 uM
PSC Rantes	-	-	1 uM
